# Multiclass classification for skin cancer profiling based on the integration of heterogeneous gene expression series

**DOI:** 10.1371/journal.pone.0196836

**Published:** 2018-05-11

**Authors:** Juan Manuel Gálvez, Daniel Castillo, Luis Javier Herrera, Belén San Román, Olga Valenzuela, Francisco Manuel Ortuño, Ignacio Rojas

**Affiliations:** 1 Department of Computer Architecture and Computer Technology, University of Granada, Granada, Spain; 2 Department of Applied Mathematics, University of Granada, Granada, Spain; 3 Center for Data Intensive Science, University of Chicago, Chicago, Illinois, United States of America; Universita degli Studi di Torino, ITALY

## Abstract

Most of the research studies developed applying microarray technology to the characterization of different pathological states of any disease may fail in reaching statistically significant results. This is largely due to the small repertoire of analysed samples, and to the limitation in the number of states or pathologies usually addressed. Moreover, the influence of potential deviations on the gene expression quantification is usually disregarded. In spite of the continuous changes in omic sciences, reflected for instance in the emergence of new Next-Generation Sequencing-related technologies, the existing availability of a vast amount of gene expression microarray datasets should be properly exploited. Therefore, this work proposes a novel methodological approach involving the integration of several heterogeneous skin cancer series, and a later multiclass classifier design. This approach is thus a way to provide the clinicians with an intelligent diagnosis support tool based on the use of a robust set of selected biomarkers, which simultaneously distinguishes among different cancer-related skin states. To achieve this, a multi-platform combination of microarray datasets from Affymetrix and Illumina manufacturers was carried out. This integration is expected to strengthen the statistical robustness of the study as well as the finding of highly-reliable skin cancer biomarkers. Specifically, the designed operation pipeline has allowed the identification of a small subset of 17 differentially expressed genes (DEGs) from which to distinguish among 7 involved skin states. These genes were obtained from the assessment of a number of potential batch effects on the gene expression data. The biological interpretation of these genes was inspected in the specific literature to understand their underlying information in relation to skin cancer. Finally, in order to assess their possible effectiveness in cancer diagnosis, a cross-validation Support Vector Machines (SVM)-based classification including feature ranking was performed. The accuracy attained exceeded the 92% in overall recognition of the 7 different cancer-related skin states. The proposed integration scheme is expected to allow the co-integration with other state-of-the-art technologies such as RNA-seq.

## Introduction

Skin cancer was predicted to account for more than a third of all cancers [1] almost two decades ago. Nowadays, this prediction is already a crude reality. Skin cancers can be classified depending on the involved cell type: keratinocytes and melanocytes. These categories are also widely known as non-melanoma skin cancer (NMSC), with a higher incidence rate, and melanoma skin cancer (MSC), with a higher mortality rate, respectively.

Within the first category, the most common skin cancer manifestation is the basal cell carcinoma (BCC). Generally, BCC almost never spreads beyond the original tumor site, rarely becoming life-threatening. However, it can be disfiguring if it is not treated promptly. The second most common form of skin cancer is the squamous cell carcinoma (SCC). It can cause death, although most cases are treatable. Both cases are followed in frequency by Merkel cell carcinoma (MCC). This subtype is considered an aggressive skin cancer with high risk of recurrence and metastasis. The MSC category represents the most dangerous form of skin cancer. In melanoma, the damaged DNA triggers mutations considered as genetic defects permitting a fast multiplication of tumoral skin cells. The early diagnosis of skin melanomas is usually determined by using ABCDE signs [2].

Notwithstanding the occurrence of skin cancer is becoming alarming, the registration standards of NMSC are highly precarious almost worldwide [3]. This is due to an insufficient data collection in cancer registries on BCC cases. Consequently, its real incidence is usually unknown [4]. Even so, skin cancer is considered the major public health problem in Australia [5, 6] and the most commonly diagnosed cancer in United States [4, 7]. Therefore, several campaigns and programs have been promoted in relation to the prevention of skin cancer in both countries [4, 8]. With respect to its incidence in Europe, NMSC has been categorized as one of the most worrying malignancies in Germany [9] as well as a systematic review reflected the current situation in Spain [10].

In order to broaden the knowledge about this disease, a wide range of machine learning (ML) and computer science approaches have been proposed: neural networks [11], image preprocessing and classification [12–14], prediction models [15, 16], pattern recognition [17], optical techniques [18, 19], etc. Although each approach focuses on improving the skin cancer diagnosis by using different techniques, a comprehensive analysis of the gene expression can extract revealing genes which could be responsible for a number of manifestations of this genetic disease [20]. In this sense, ML techniques efficiently help to select those genes with the highest informative power for the diagnosis.

The current trends of high-throughput gene expression analysis lead to use RNA-seq instead of microarrays, mainly due to its multiple advantages [21]: i) RNA-seq allows detecting the variation of a single nucleotide; ii) it does not need genomic sequence knowledge; iii) it provides quantitative expression levels and isoform-level expression measurements; and finally, iv) it offers a broader dynamic range than microarrays. However, even though the imposition of RNA-seq is a matter of time, microarrays still have many factors in their favor. Above all, microarrays have been used so far, and are still in use, because they are cheaper. Consequently, a wider number of datasets are available at the moment what has led to the publication of many skin cancer studies in the last years [22–25].

Nonetheless, these researches are often conducted on a limited sample set, thus reducing the chance of reaching statistically significant results. Similarly, the number of pathological states of skin cancer analysed is reduced, thereby obtaining different DEGs sets by using traditionally binary classifiers for each isolated experiment. As a solution to these limitations, collecting different datasets including skin cancer samples of diverse pathological states from various experiments, may considerably increase the robustness of the study and help in identifying biomarkers for the differentiation of a wider range of pathological states. However, the joint consideration of cancer datasets with different technical characteristics usually involves dealing with the removal of batch effects which must be taken into account for an effective integration [26].

Multiclass classification has been approached for a wide range of cancerous diseases in several previous works (breast [27], colorectal [28], ovarian [29], prostate [30], etc.). Specifically, a number of skin cancer studies have used this strategy from the analysis of histopathological [31, 32] or dermoscopic images [33–36]. However, this cancerous disease have not practically addressed from the analysis of gene expression at the multiclass level. Hierarchical clustering has been exclusively used in order to compare gene expression signatures from different skin pathological states [37]. Based on the use of highly-discriminant DEGs, any new patient skin sample could be assessed and correctly classified by distinguishing among several skin pathological states in a single analysis [38]. Since the cancer prognosis is much more encouraging when a patient diagnosis is available at an early stage, clinicians can take advantage of relying the final diagnosis on its assessment [39]. Consequently, at the dawn of the personalized medicine, predisposition to certain skin cancer manifestations could be properly detected [40], and unnecessary medical treatments such as radiation therapies, excision surgeries or medications supply could be prevented [41].

Under all these considerations, a novel skin cancer diagnosis approach based on the integration of multiple skin cancer datasets from several microarray platforms is presented in this work. Up to our best knowledge, the integration of different datasets based on gene expression analysis still remains unprecedented. Firstly, an exhaustive data acquisition about patient and control skin samples has been performed from NCBI GEO web platform [42, 43]. A total of 24 series containing 770 microarray samples were collected in first instance. However, only 678 of them finally passed the quality control and were subjected to the pre-processing phase: 554 samples from Affymetrix platforms [44] and 124 samples from Illumina platforms [45]. Next, the integration is carried out by using a well-known tool for samples fusion (virtualArray). These DEGs were obtained after several statistical restrictions and fusion decisions in the presence of diverse factors such as batch effects. Under its operation, 17 DEGs were preselected as reliable candidate biomarkers of the most relevant skin cancer pathological states. An ANOVA statistical test validated these DEGs as powerfully informative and statistically differentiated. Subsequently, in order to identify and assess the most outstanding genes, a SVM classifier was applied in association with a minimum-Redundancy Maximum-Relevance (mRMR) feature selection algorithm. The biological interpretation of the selected genes was finally contrasted using the specific literature and it is included in detail as an appendix in this work.

The structure of this paper can be summarized as follows: this section motivated and introduced the present work. Next section explains the materials and methods used in this study to construct the presented pipeline. Following, the results section shows the applied data processing. Firstly, the number of samples selected after the quality control and the number of genes obtained after the integration. Consequently, the analysis of the integrated gene expression, revealing those genes which remain unchanged regardless of union and batch effect removal methods. This section ends with the presentation of the classification tool results. Subsequently, the discussion section comments on the obtained results as well as on the contributions of this study. Finally, the conclusions section underlines the validity of the proposed approach, its utility for early diagnosis and medical support, as well as the future work. Among the following purposes, it is expected the integration with other omics technologies such as RNA-seq and the application to a wide range of other human pathologies.

## Materials and methods

### Samples

All analysed RNA samples were obtained from NCBI GEO web platform. An exhaustive search was carried out covering the main skin cancerous manifestations for which registers were found in this public database. The two most well-known microarray technologies (Illumina and Affymetrix) were considered for this purpose. Thus skin carcinoma, skin melanoma and healthy skin categories were finally chosen. The first category is comprised by the NMSC variants already mentioned in the introduction section: BCC, SCC and MCC samples. The next category collects melanoma samples, distinguishing between two types: primary melanoma and metastatic melanoma. Both melanomas have been labeled as PRIMEL and METMEL, respectively. The last category includes samples from healthy skin, differentiating between skin samples with and without nevus. In this case, these samples were marked as NEV and NSK. Other important cancer manifestations such as Langerhans cells, among others, were not considered as no registers were found in the database.

Under the specified operation framework, 24 series from Affymetrix and Illumina platforms were selected. These series are publicly available at https://www.ncbi.nlm.nih.gov/geo/query/acc.cgi?acc=S.NAME where S.NAME is the name of each series at NCBI GEO web platform. Table 1 summarizes the information about the series before and after the quality control phase. More details about the distribution of the skin samples for each microarray series used in this work is specified in S1 Appendix.

**Table 1:** NCBI GEO series selected for this study. Criteria for series selection was getting a relative balancing of the different categories, including all possible samples from the least frequent diseases. Technology and total number of samples/outliers are included.

Series	Technology	Samples origin	Skin states ordered by frequency (*)	# High quality samples	# Excluded outliers
GSE2503	Affymetrix	Berlin (Deutschland)	SCC, NSK	10	1
GSE3189	Affymetrix	San Diego (USA)	PRIMEL, NEV, NSK	66	4
GSE6710	Affymetrix	Berlin (Deutschland)	NSK	12	1
GSE7553	Affymetrix	Tampa (USA)	BCC, PRIMEL, SCC, NSK	44	2
GSE13355	Affymetrix	Ann Arbor (USA)	NSK	57	7
GSE14905	Affymetrix	Gaithersburg (USA)	NSK	20	1
GSE15605	Affymetrix	Nashville (USA)	PRIMEL, NSK, METMEL	46	18
GSE29359	Illumina	New Lambton Heights (Australia)	METMEL	75	7
GSE30999	Affymetrix	Spring House (USA)	NSK	74	11
GSE32407	Affymetrix	New York (USA)	NSK	10	0
GSE32628	Illumina	Leiden (Netherlands)	SCC	14	1
GSE32924	Affymetrix	New York (USA)	NSK	7	1
GSE36150	Affymetrix	Royal Oak (USA)	MCC	10	5
GSE39612	Affymetrix	Ann Arbor (USA)	MCC, SCC, BCC	28	12
GSE42109	Affymetrix	New York (USA)	BCC	10	1
GSE42677	Affymetrix	New York (USA)	SCC	10	0
GSE45216	Affymetrix	London (United Kingdom)	SCC	28	2
GSE46517	Affymetrix	Houston (USA)	METMEL, PRIMEL, NSK, NEV	78	10
GSE52471	Affymetrix	New York (USA)	NSK	10	3
GSE53223	Affymetrix	New York (USA)	NEV, NSK	14	4
GSE53462	Illumina	Suwon (South Korea)	BCC, SCC, NSK	25	1
GSE55664	Illumina	Philadelphia (USA)	NSK	10	0
GSE66359	Affymetrix	Turku (Finland)	SCC	8	0
GSE82105	Affymetrix	New York (USA)	METMEL, NSK	12	0
TOTAL	Integrated			678	92

(*) Skin states of each series are ordered from most frequent to the lowest frequent one

From this selection of RNA samples, the following taxonomies were proposed (see Table 2 for complete information including number of samples for each category):

- tumor and healthy samples as the most general taxonomy (2 classes taxonomy).
- carcinoma, melanoma and healthy samples (3 classes taxonomy).
- BCC, SCC, MCC, PRIMEL, METMEL, NSK and NEV samples (7 classes taxonomy).

**Table 2:** Taxonomic classifications for the three skin cancer scenarios: 2, 3 & 7 classes.

	Carcinoma (NMSC)			Melanoma (MSC)		Healthy Skin	
	BCC	SCC	MCC	PRIMEL	METMEL	NSK	NEV
7 classes	43	84	33	118	118	250	32
3 classes	160			236		282	
2 classes	396						
TOTAL	678						

### Tools

R [46] and MATLAB [47] programming languages were used in this study. Most of the used R packages derived from Bioconductor platform [48]. This platform is an open-source and open-development software built in the R statistical programming environment for the analysis and comprehension of genomic data. The tools contained in the Bioconductor project represent many state-of-the-art methods for the analysis of microarray and genomic data. Other R packages come from CRAN [49], a network of ftp and web servers around the world storing identical, up-to-date versions of code and documentation for R.

### Pipeline

**Fig 1:**
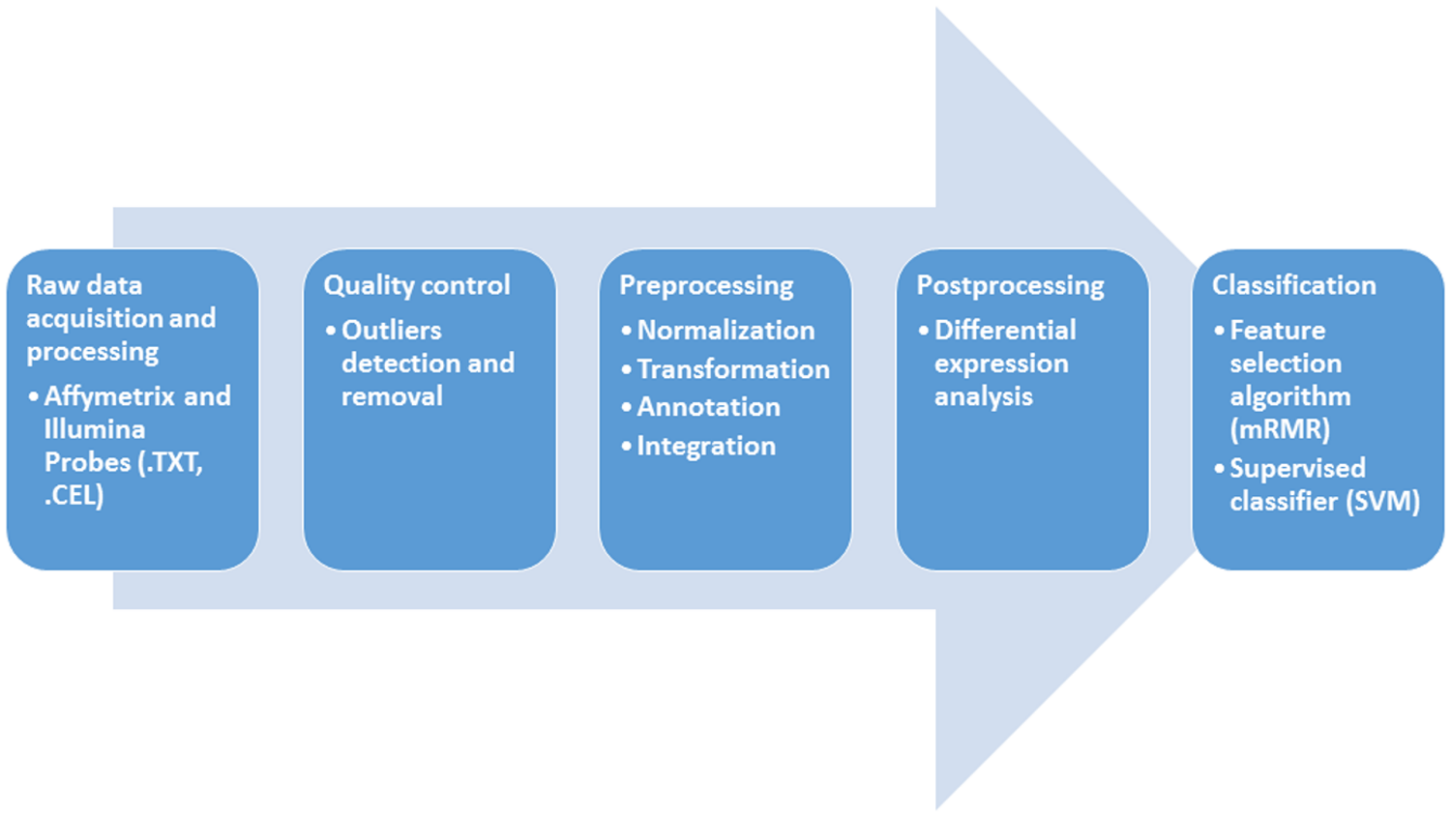
Microarray gene expression analysis pipeline. The process has been developed sequentially in different phases. This pipeline summarizes the decisions made throughout the study.

Our work has been based on the steps specified in Fig 1. Each one of the phases carried out is explained in the next subsections.

#### Raw data acquisition and preparation

Acquiring raw data is the very first step in any analysis. Each vendor quantifies its raw data in a different format, even with different platforms. Therefore, a particular procedure has to be applied for each series. In this study, several R packages have been used to download the microarray datasets in a programmatic manner. The Bioconductor *affy* package was used to read and process Affymetrix CEL files for their later preprocessing [50]. *GEOquery* package [51] was necessary in order to obtain already preprocessed RNA samples (when RNA samples CEL files are not available). For the newer Affymetrix microarrays, the Bioconductor *oligo* package [52] was employed. For the Illumina microarrays, the *lumi* package [53] has been used.

#### Quality control

Assessing the quality of the experiments is an essential step in microarray analysis as array-based technologies present inherent biases. Bioconductor *arrayQualityMetrics* package [54] is widely used for chip analysis and its use is not limited to one technology. It provides tests that consider quality metrics over the series samples as comparisons, intensity distributions, variance mean dependence and individual quality, for the detection of samples with insufficient quality (outliers). These tests include: distance among samples, principal component analysis (PCA), Kolmogorov-Smirnov test based on the *K*_*a*_ parameter, density distribution plots, standard deviation of the samples intensities and Hoeffding’s D-statistic (normally executed with D < 0.15). All of them are iteratively applied over a given series until outliers are no longer detected or considered. Final number of excluded outliers from the considered series is shown in the last column of Table 1.

#### Preprocessing

Applying a preprocessing step on microarray data is crucial, especially when different platforms and technologies are integrated. More specifically, microarray technologies usually require normalization, which involves a platform-dependent process necessary for converting raw data probe intensities into expression values. In this study, the Robust Multi-array Average (RMA) algorithm [55] was applied on the collected microarray data. RMA performs background correction, normalization, and summarization in a modular way. For Affymetrix microarrays, it can be achieved by means of the *rma* function from *affy* and *oligo* packages. In the case of Illumina microarrays analysis, the homologous *lumiExpresso* function from *lumi* package was used, allowing to do all processing steps simultaneously.

After microarray normalization, other factors have to be taken into account for a correct microarray integration. On one hand, the logarithmic transformation must be done on the different series as well as the bit depth homogenization. Both processes are necessary in order to avoid scale errors in further analysis. In particular, all series required logarithmic transformation in base 2. However, only 4 series had to be changed to 16-bit depth: GSE2503, GSE3189, GSE29359 and GSE55664. This type of transformations should be applied to any new sample before it can be classified correctly through the pipeline proposed in this study.

A final verification of correct series annotation was made by checking annotation data for different chips from Bioconductor AnnotationData Packages website. The main reason lies in avoiding further integration errors. They can likely come from either a missing annotation in the raw data taken from NCBI GEO web platform or after the application of the previous pre-processing R routines. Table 3 summarizes different R packages of annotation data chips included in this work.

**Table 3:** Bioconductor R AnnotationData packages and available symbols for the selected series integration.

Annotation data chip	# Platform	# Possible symbols	# Symbols with annotation	# Symbols NA’s	# Symbols integrated by virtualArray	# Series
hgu133a	GPL96	24549	23392	1157	12442	4
hgu133a2	GPL571	24543	23390	1153	12441	4
hgu133plus2	GPL570	58616	48709	9907	20545	11
huex10sttranscriptcluster	GPL5175	26387	21988	4399	15016	1
illuminaHumanv2	GPL6104	22916	21965	951	17296	1
illuminaHumanv4	GPL10558	50613	39181	11432	21035	1
lumiHumanAll	GPL6102	47323	31403	15920	20787	2
TOTAL (Integrated Symbols)					9978	24

Different number of symbols were achieved depending on the platform and technology employed. NA ≡ Not Available.

Finally, the sample integration is possible by means of packages as virtualArray [56], readbulk [57] or inSilicoMerging [58] in association with inSilicoDb [59]. These tools have in common that allow combining multiple microarray samples with different strategies, but not all have the necessary characteristics for this study. While readbulk can only collect heterogeneous datasets, inSilicoMerging only works with Affymetrix platforms. This last package can also normalize and remove batch effect over multiple datasets of Affymetrix technology, but virtualArray package allows merging additional datasets from other technologies as Illumina. For this reason, the package virtualArray was chosen for this approach.

Additionally, the impact of two factors on the quantification of genes can be evaluated with this tool: batch effect and union method. The first one takes into account the variations in gene expression due to biological, technical and even atmospheric agents [60]. Taking into account the hypothetical influence of this factor is considered as a compulsory step in any study of high-throughput data [61]. Currently, dealing with it is becoming challenging because there is no absolute certainty about removing the batch effects even after applying correction algorithms. An effective removal may be essential for effective integration of different datasets [26]. In this sense, the virtualArray package allows evaluating up to 6 different batch effects without losing biological information on the quantification of the gene expression: GQ [62], EB [63], NORDI [64], QD [62], MRS [62] and MC [65]. The second one allows summarising in a single value all the values of expression of genes that transcribe the same gene identifier. All transcripts can be gathered into a single expression value in order to be consistent in evaluating the impact of each gene selected in the study. To evaluate its effect, this tool allows 2 union methods: mean and median. Therefore, and in search of independence in the process, a total of 12 configurations from the combination between the 6 batch effects and the 2 union methods have been tested. Consequently, only those genes that are also robust to these factors are obtained.

#### Post-processing

The next step in the microarray analysis methodology is calculating and obtaining DEGs. In our work, a seven-classes taxonomy was considered for DEGs identification. Then, those results were translated to the three-classes and the two-classes taxonomies for assessment.

The *limma* package [66] is commonly used since it includes interesting supplementary features: in addition to calculating DEGs, it allows making heatmaps and Venn diagrams. Although there are several statistical parameters that are taken into account in this type of studies as moderated t-statistic (T) or B-statistic (B), special attention was paid to other two parameters: log-fold change (LFC) and p-value (PV). Restrictive values for those two parameters were considered in order to guarantee statistically highly differentiated candidate genes.

This decision is motivated by the fact that certain variations can be expected among the quantification values of the genes since data are being taken from different platforms. Because of this, they could influence the selection of the genes that define the considered skin states. To avoid the potential influence of these factors, it is important to impose severe statistical restriction values on these parameters with the aim of taking those genes that are as representative as possible.

With these premises, each configuration was subjected to evaluation from the imposition of the finally chosen values for LFC and PV of 4 and 0.001, respectively. Then, a joint result was obtained, by selecting as definitive candidates based on the matches among those configurations returning candidates.

Once a set of genes has been selected, it is very important to know the robustness of the expression of these DEGs when processing microarrays from different technologies. From this perspective, the main goal is to analyze whether the variation in the expression of these DEGs is mainly due to the different cancer-related skin states considered in this study or there are also other relevant factors involved in the processing (such as the batch effect, the country of origin of the samples or the union methods considered). In order to perform a statistical analysis that can encompass the information of all DEGs simultaneously, a dependent variable has been designed based on the Least Squares concept [67]. This algorithm takes into account the difference between the expression value of each of the candidate genes and their mean over all experiments and preprocessing variants. An ANOVA statistical test [68] was performed in order to verify the robustness of the selected genes with respect to a number of factors: “country”, “type” (7 cancer-related skin states), “batch effect” and “union method”. This test allowed us to confirm the study feasibility and robustness, in the selection of the identified skin cancer biomarkers.

Finally, after all the post-processing tasks were performed, the DEGs identified by the proposed methodology were consulted in different databases in order to assess their hypothetical relationship with skin cancer. DisGeNET [69], WikiGenes [70], DISEASES [71] and Open Targets [72] databases were employed for this purpose. Additionally, a text mining tool, “Gene Set to Disease” (GS2D) [73], was applied to extract the relation among the DEGs with skin diseases or disorders.

#### Classification

The traditional microarray data processing typically ends with the determination of DEGs. The experts can usually check these highlighted genes with laboratory experimentation or contrast them with past works. However, a great interest is aroused in relation to which DEGs are more relevant according to the analysed data groups.

This work moves one step ahead by applying ML techniques in order to gain knowledge on the relevance of the selected genes. Similarly, a classification model is designed to automatically classify new data samples.

With the objective of discerning among the involved seven cancer-related skin states, a ranking of the most significant DEGs was obtained by using the well-known and effective mRMR algorithm [74]. This algorithm takes into account the redundancy contained among the considered genes, identifying the genes that add complementary information. This leads to attaining simpler classifiers with lower number of genes. The mRMR algorithm made use of the Kraskov Mutual Information estimator [75].

The classification technique considered in this work is the SVMs [76]. Then, two cross-validation techniques were applied to assess the classifier performance: Leave-One-Out cross-validation (LOO-CV) [77] and K-Fold cross-validation (KFOLD-CV, where K = 10) [78].

## Results

### Biological samples integration

24 series from Affymetrix and Illumina platforms were selected. Table 1 summarized the series selection process and the samples relevant to the study. 92 RNA samples were considered outliers and discarded after a strict quality control from the initial selection of 770 RNA samples.

**Fig 2:**
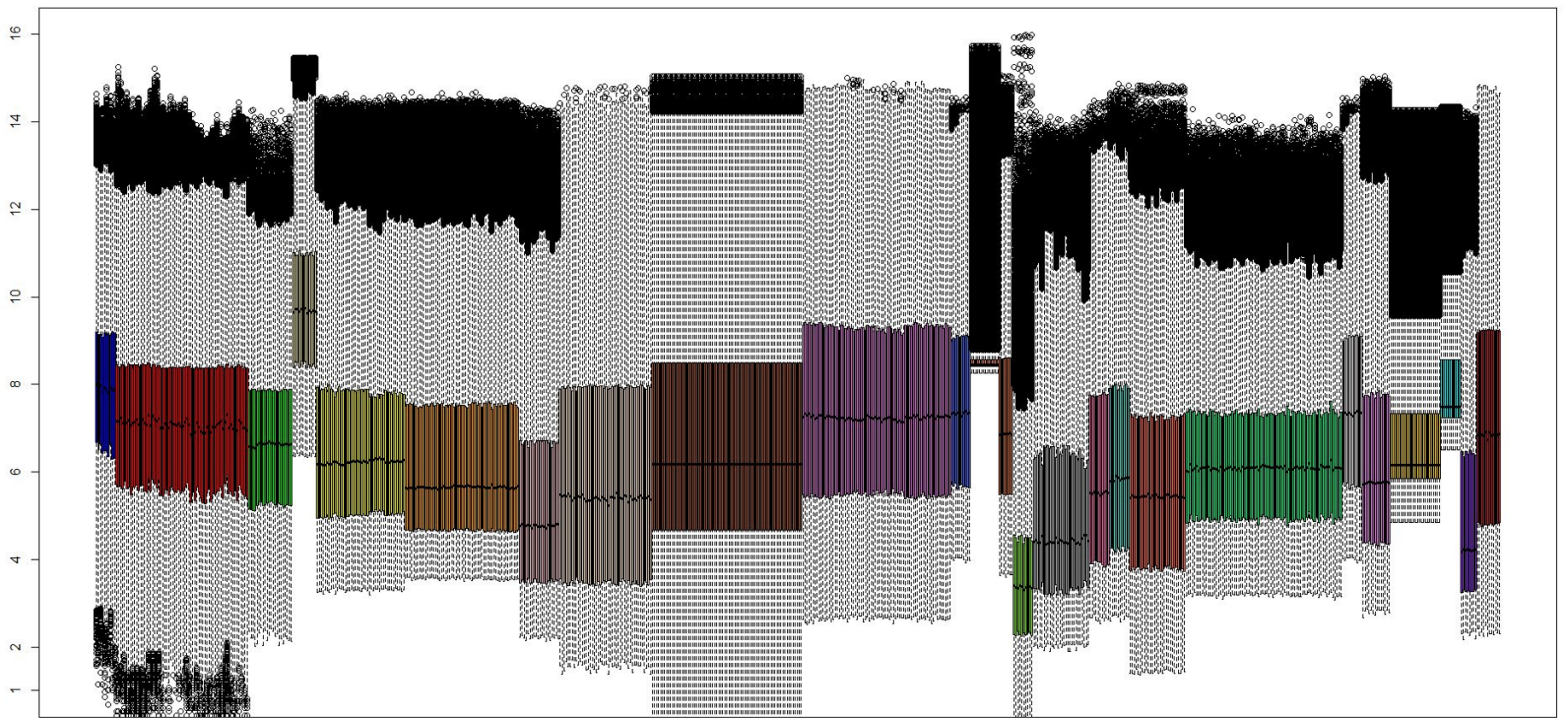
Expression values of each series after independent normalization. The aggregation of the high quality samples shows dynamic variability among different datasets.

**Fig 3:**
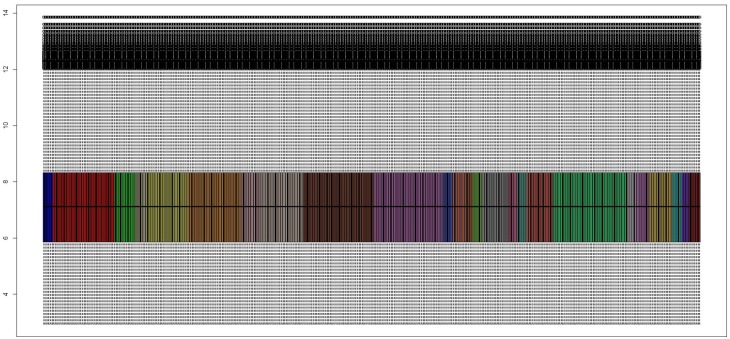
Expression values of each series after joint platforms normalization. The integration tool used on the high quality samples reflects a homogeneous expression range.

The joint representation of individual series normalization reflected several expression value ranges (Fig 2). An additional preprocessing was carried out by using virtualArray tool in order to remove the samples dynamic variability, so that a homogeneous expression range was obtained for 678 high quality RNA samples (Fig 3).

12 different configurations, coming from six batch effects using two different union methods, were applied through this tool on all 678 RNA samples. This was made in order to invalidate the influence of intrinsic anomalies on the quantification of the genes. A cross-platform normalization and batch effect removal was simultaneously applied. Regardless of the configuration applied, 9978 genes were coerced through correct annotation (Table 3).

### Expressed genes selection

As several heterogeneous data series were put together, and with the aim of attaining statistical robustness in the selection of DEGs, all possible batch effect validations provided by virtualArray package were tested. Similarly, strong conditions were imposed to the statistical parameters involved. Values of |*LFC*| ≥ 4, PV ≤ 0.001 were finally selected. Table 4 summarizes the number of expressed genes after evaluating each of the 12 configurations.

**Table 4:** Total number of obtained DEGs depending on several restrictions imposed by different evaluated configurations of virtualArray tool. The batch effect removal and union method factors were considered. The statistical parameters |*LFC*| ≥ 4 and PV ≤ 0.001 were selected.

Batch Effect	GQ	QD	EB	NORDI	MRS	MC
Union Method						
Mean	0	25	0	0	39	39
Median	0	23	0	0	41	41

**Fig 4:**
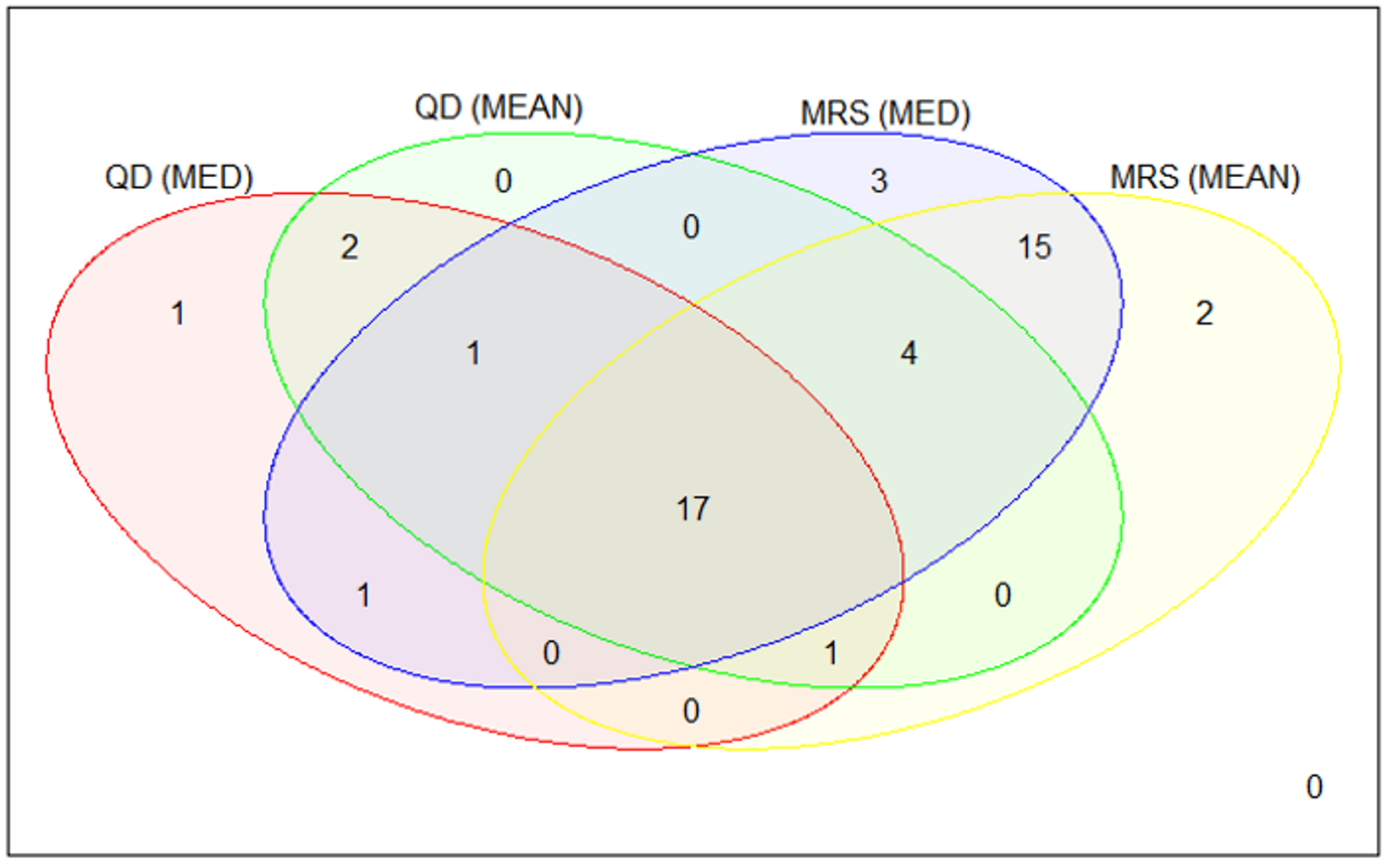
Final common DEGs obtained by considering common genes from QD and MRS results intersection. 17 common DEGs were obtained between QD and MRS effect batch removal in addition to apply union methods intersection.

DEGs appearing in several of the configuration outcomes were expected to perform robustly as potential biomarkers of skin cancer. Therefore, the intersection of candidate DEGs for configurations QD and MRS by using both union methods (configuration MC got the same results as MRS) was carried out. This guarantees that possible anomalies, coming from the heterogeneous union of datasets, would have no effect on the discriminative gene selection. The Venn diagram in Fig 4 shows the common DEGs among the 4 considered configurations. Resulting DEGs selected from this intersection are shown in Table 5; it includes the main statistical parameters presented by limma package in a summary way. Average and standard deviation values for LFC, T and B parameters were included considering the cases in where required statistical restrictions were fulfilled. Also, minimum and maximum PV were specified for these cases. Additionally, in the DEG cases column, the number of times each gene is present as a DEG is given. This number is calculated doing pair comparisons between two classes which results in a total of 21 pair comparisons taking into account the 7 cancer-related skin states.

**Table 5:** List of 17 DEGs which are independent to the union method, batch removal method and multiclass problem. One of the virtualArray configurations (union method by mean, MRS batch effect and 7 classes taxonomy) was selected for showing those DEGs. All of them were listed and ordered by |*μ*_*LFC*_|.

Gene Symbol	# DEG cases	|*μ*_*LFC*_ ± *σ*_*LFC*_|	|*μ*_*T*_ ± *σ*_*T*_|	[*PV*_*min*_, *PV*_*max*_]	|*μ*_*B*_ ± *σ*_*B*_|	Related to skin cancer
PCP4	5	6,4649 ± 0,7160	29,8327 ± 5,3161	[1, 37E-147, 2, 34E-75]	275,6113 ± 68,3340	No
TYRP1	5	5,8649 ± 0,9581	13,3323 ± 2,3796	[4,06E-47, 4,29E-23]	72,3469 ± 24,6074	Yes (DisGeNET)
ISL1	6	5,7691 ± 0,4387	24,3430 ± 1,9227	[5,16E-106, 1,78E-76]	204,9844 ± 24,8309	Yes (TargetValidation)
POU4F1	6	5,6337 ± 0,5265	30,5412 ± 3,4649	[2,94E-164, 2,93E-112]	284,6693 ± 43,9275	Yes (DISEASES)
DSC3	8	5,3559 ± 0,4448	22,3521 ± 8,3026	[6,89E-179, 1,65E-41]	180,4227 ± 104,3494	Yes (WikiGenes)
DSC1	9	5,3304 ± 0,5153	16,3742 ± 5,0402	[3,20E-111, 7,55E-27]	108,0049 ± 60,6726	Yes (DisGeNET)
MLANA	8	5,3174 ± 1,1594	19,0411 ± 4,0277	[3,02E-94, 1,48E-30]	138,9094 ± 48,6644	Yes (DISEASES)
SOSTDC1	3	5,0053 ± 0,4610	18,9934 ± 1,1818	[8,34E-70, 1,05E-57]	136,8830 ± 14,5191	No (*)
TGM3	2	4,9463 ± 0,5166	19,9389 ± 1,6022	[3,32E-76, 8,20E-64]	148,6677 ± 20,0540	Yes (TargetValidation)
CLDN1	6	4,8115 ± 0,5509	17,3556 ± 1,1748	[1,16E-63, 3,65E-48]	116,9768 ± 14,0670	Yes (TargetValidation)
MYO15A	6	4,7396 ± 0,4262	33,1131 ± 5,4205	[3,90E-178, 1,70E-101]	316,6801 ± 68,1730	No
BNC2	2	4,7061 ± 0,0239	24,5090 ± 3,5099	[1,56E-109, 1,60E-81]	206,8020 ± 44,9266	Yes (DISEASES)
SCGB2A1	5	4,6701 ± 0,3740	16,6978 ± 2,7768	[6,47E-68, 4,84E-32]	110,0158 ± 31,7911	No (*)
CRYBA2	5	4,6490 ± 0,2739	33,2294 ± 4,2398	[9,09E-172, 3,93E-117]	318,5401 ± 53,3786	No
ANXA3	2	4,5959 ± 0,1186	17,7576 ± 1,0363	[4,05E-62, 2,21E-54]	121,7490 ± 12,5127	No
KRT20	6	4,5916 ± 0,2552	27,1811 ± 3,9432	[7,86E-142, 3,50E-77]	241,6544 ± 50,8763	Yes (TargetValidation)
LGR5	1	4,3562 ± 0,0000	21,5629 ± 0,0000	6,22E-79	169,1057 ± 0,0000	Yes (DisGeNET)

(*) Related to epithelial tissues

The ANOVA test allowed determining the influence of different factors considered on the 17 expressed common genes quantified and extracted from different microarrays. From its assessment, the analysed cancer-related skin state has been showed to be the factor with greater repercussion on the variation in the expression of such genes. Therefore, these 17 expressed common genes were cataloged as hopeful candidates for skin cancer biomarkers. Also, these genes are able to discern as much as possible among the seven skin states considered in this study. In accordance with this, Table 6 summarized the main statistics parameters of this analysis and supported the independent selection of any configuration for the subsequent analysis of the 17 DEGs quantification values. More information about the carried out ANOVA test can be consulted in S2 Appendix.

**Table 6:** Results of the ANOVA test. The statistical analysis includes the main factors assessed, such as relevant statistics parameters among which highlights associated PV.

Source (Main Factors)	Sum of Squares	Df	Mean Square	F-Ratio	P-Value
A: TYPE (*)	6,28026	6	1,04671	1521,63	*0,0000*
B: BATCH (*)	1,51534	1	1,51534	2202,88	*0,0000*
C: METHOD	0,00112746	1	0,00112746	1,64	**0,2005**
D: COUNTRY (*)	0,332589	6	0,0554316	80,58	*0,0000*
RESIDUAL	1,85524	2697	0,000687889		
TOTAL (CORRECTED)	10,1783	2711			

(*) Statistically significant factors

### Gene set assessment & hierarchical clustering

**Fig 5:**
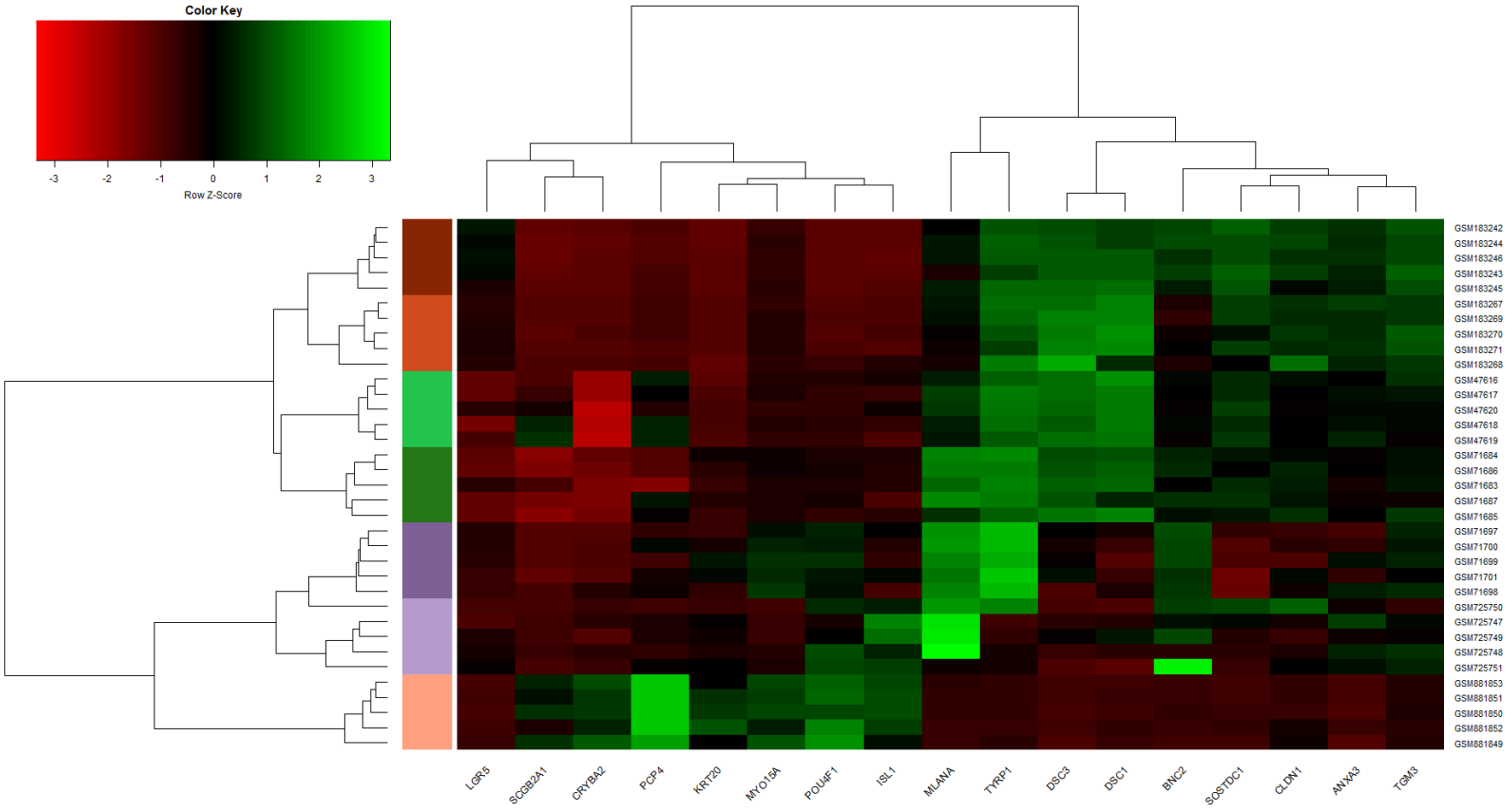
Hierarchical clustering of healthy and skin cancer samples by using the 17 DEGs. A perfect differentiation among the 7 cancer-related skin states was obtained after applying clustering and dendrogram reorder. Five samples from each skin state were used. Different colors are used for each skin sample type: NSK (light green), NEV (dark green), PRIMEL (dark purple), METMEL (light purple), BCC (chocolate), SCC (orange) and MCC (salmon).

With the aim of illustrating the joint discriminatory power of the 17 DEGs analysed in this study, a hierarchical clustering of a selection of samples from each skin state is presented in Fig 5. A suitable cluster separation and a inter-cluster grouping among similar cancer-related skin states were achieved thanks to the dendrogram reorder performed by using the Ward’s method [79]. On the top, both skin carcinomas (BCC and SCC) were put together. Next, both healthy skin states (NSK and NEV) and both skin melanoma states (PRIMEL and METMEL) were sequentially listed. At the bottom, MCC was separated from the other skin carcinomas as it practically exhibits opposite expression values for almost all the selected genes. In the light of all this, the different selected genes show to have an expectable remarkable discriminative power to differentiate among the different cancer-related skin states as well as to obtain a reliable skin cancer diagnosis.

### Gene relevance identification & classification process

An assessment of the quality of the information provided by the 17 validated DEGs is necessary in order to reduce the complexity of the study. It also allows to limit the effective diagnostic potential of skin cancer to only a small set of genes.

Different databases were consulted with the aim of checking the relationship between these genes and skin cancer. Table 5 points out if the identified DEGs were previously reported as related to the cancer-related skin states, according to the consulted databases. Full and exhaustive information about the biological relationship of these genes with skin cancer and other cancers can be seen in S3 Appendix.

**Fig 6:**
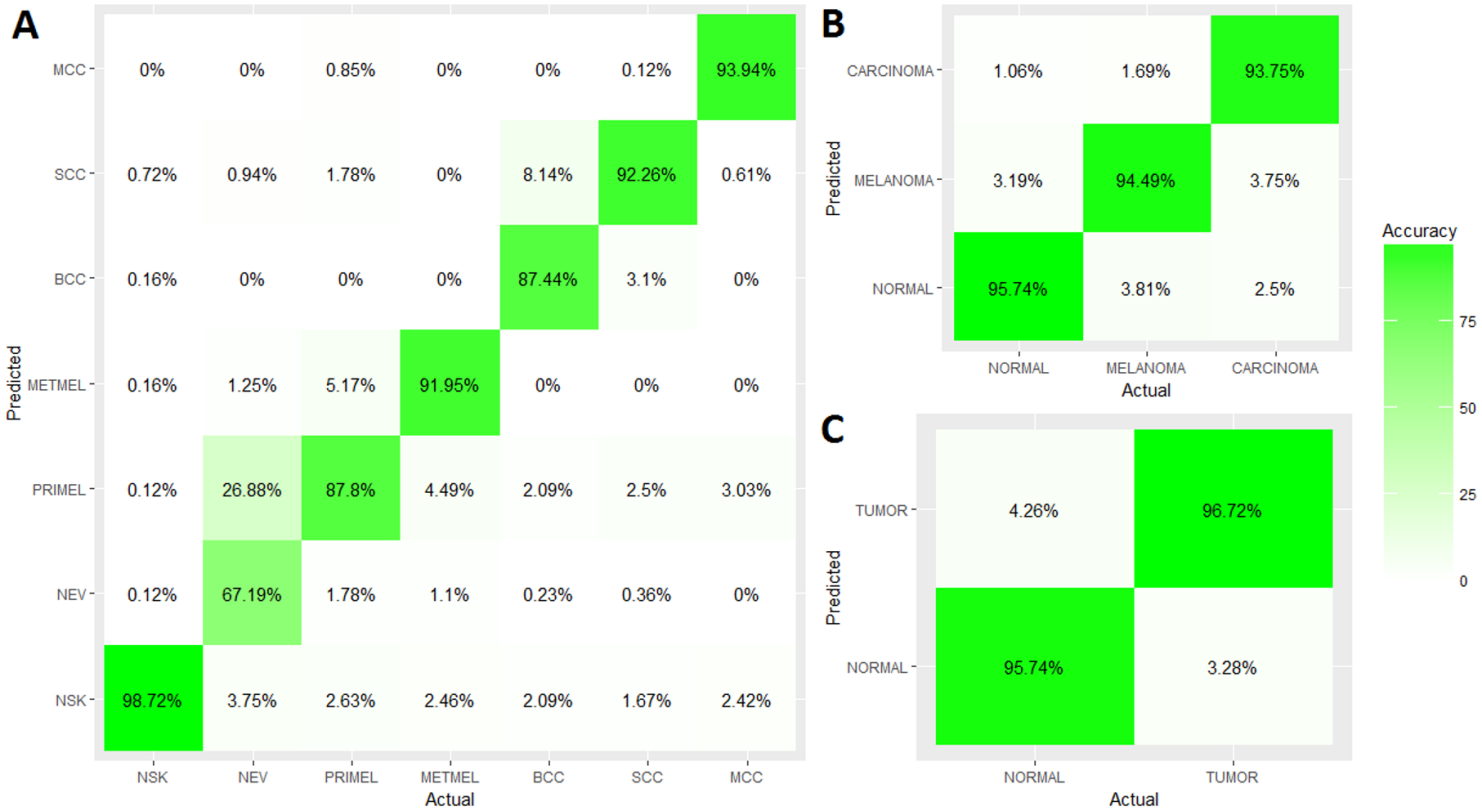
Classification accuracy achieved for each of the considered taxonomies: (A) 7 classes, (B) 3 classes and (C) 2 classes. The confusion matrix for taxonomy A was constructed with 10-CV and 17 DEGs. The other confusion matrices were constructed from the previous, by summing the respective sub-matrices associated with each skin super-state.

In order to assess a hypothetical classification procedure, special precaution must be taken regarding the information provided by the selected set of genes in a new skin sample. For this reason, a classification model based on SVM multiclass was designed together with two cross-validation processes (LOO and 10-FOLD) for its assessment. The results reflect an overall accuracy recognition for the 7 cancer-related skin states considered up to 92% for both cross-validation processes. Translating this percentage into the 2 additional taxonomies considered of 3 classes (melanoma, carcinoma and healthy skin) and 2 classes (tumoral and healthy skin), this percentage increased to 95% and 96%, respectively. The associated confusion matrices can be seen in the Fig 6.

**Fig 7:**
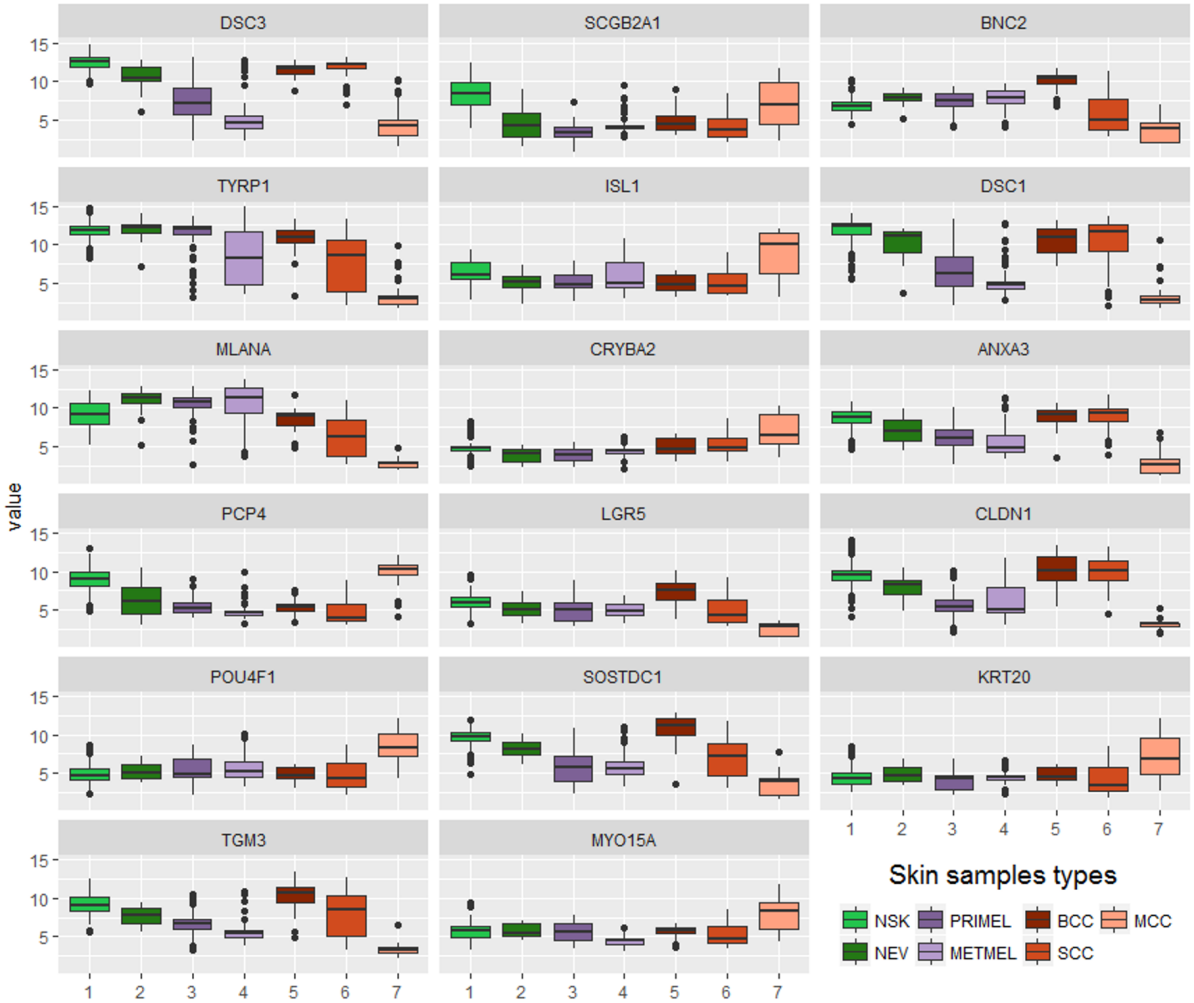
Expression level of the selected genes ordered by the ranking returned by mRMR algorithm. Different colors are used for each cancer-related skin state: NSK (Normal Skin), NEV (Nevus), PRIMEL (Primary Melanoma), METMEL (Metastatic Melanoma), BCC (Basal Cell Carcinoma), SCC (Squamous Cell Carcinoma) and MCC (Merkel Cell Carcinoma).

This previous result does not allow appreciating objectively the informative contribution of each gene to the skin state recognition. For this reason, the mRMR algorithm was employed in order to obtain a ranking of these genes according to their potential in the seven skin states discernment. The genes ranking returned by the algorithm is as follows: DSC3, SCGB2A1, BNC2, TYRP1, ISL1, DSC1, MLANA, CRYBA2, ANXA3, PCP4, LGR5, CLDN1, POU4F1, SOSTDC1, KRT20, TGM3 and MYO15A. The expression value distribution of each selected gene sorted by this ranking over each of the cancer-related skin states can be seen in the Fig 7.

**Fig 8:**
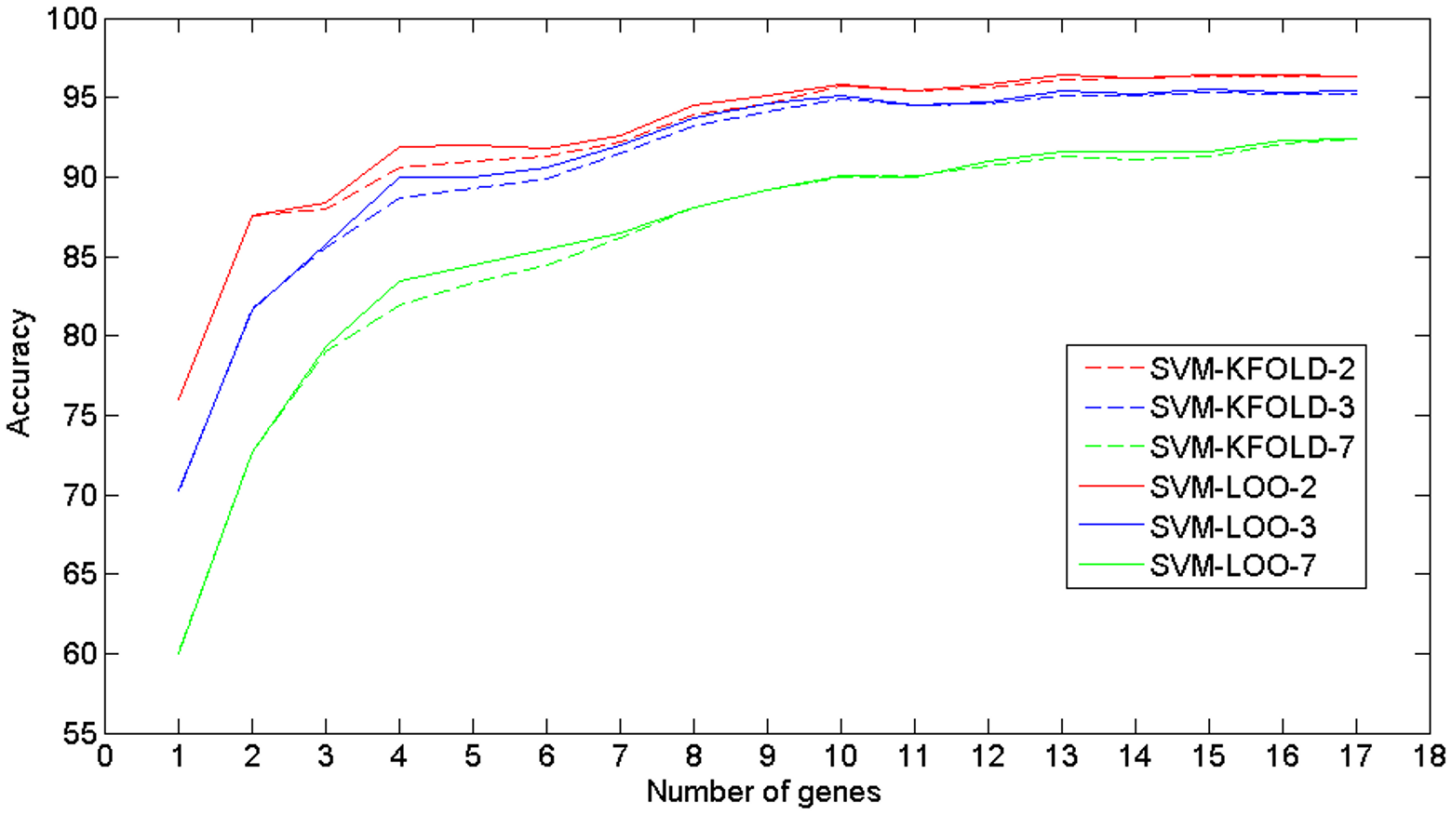
Evolution of the classification accuracy for each subset of genes considered, and for each taxonomy. Similar trends can be observed for both LOO-CV and 10-CV.

**Fig 9:**
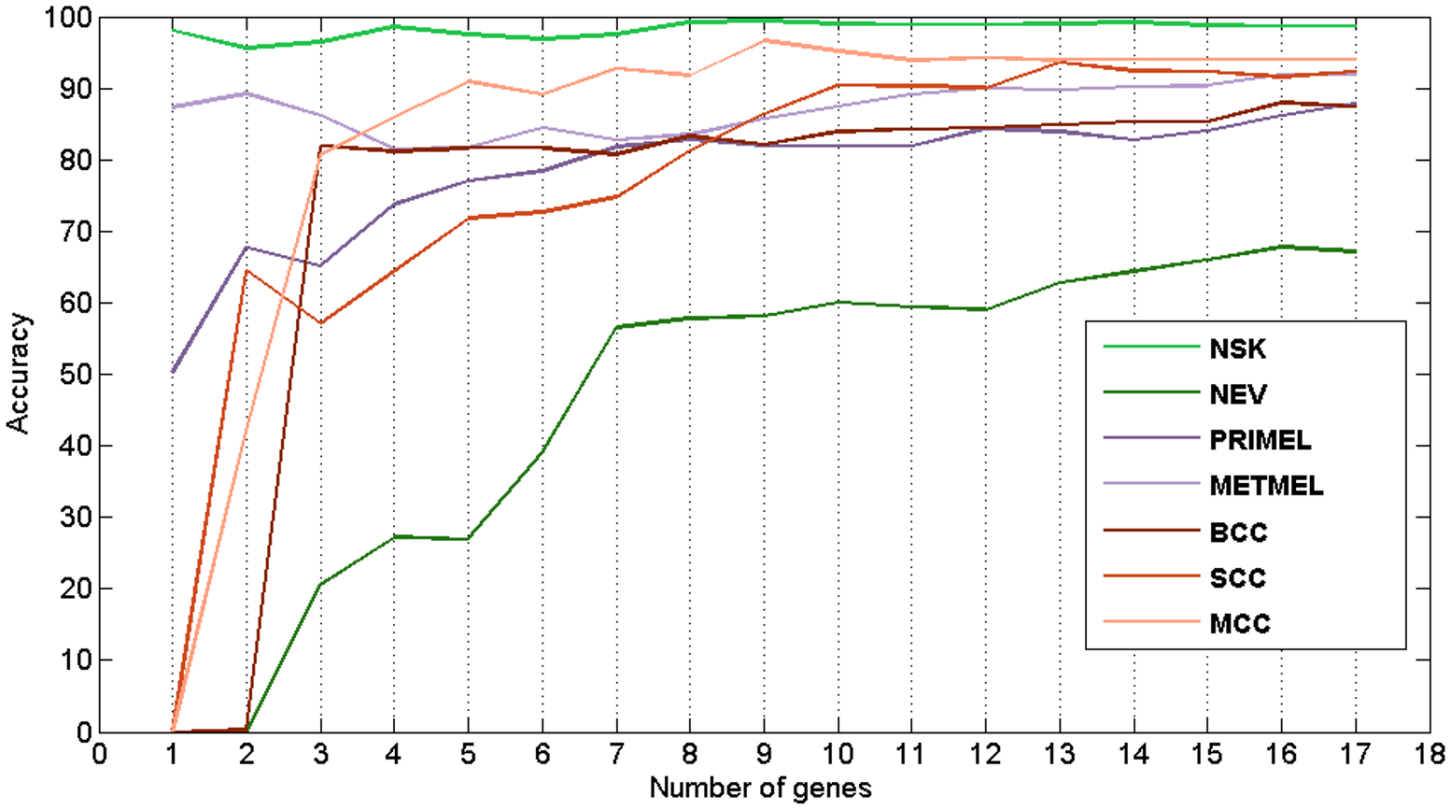
Evolution of the classification accuracy for each cancer-related skin state according to the number of genes from the mRMR ranking considered in the classifier. Different colors are used for each skin sample type: NSK (Normal Skin), NEV (Nevus), PRIMEL (Primary Melanoma), METMEL (Metastatic Melanoma), BCC (Basal Cell Carcinoma), SCC (Squamous Cell Carcinoma) and MCC (Merkel Cell Carcinoma). SVM with 10-CV was used.

Next, distinct SVM models were designed and retested by cross-validation processes in order to assess the classification capacity of different subgroups of genes returned by this ranking. The gene ranking classification results on the three considered taxonomies can be seen in the Fig 8. Finally, an evaluation of the designed classifiers behaviour was carried out for each of the cancer-related skin states. The accuracy results for each skin state and for each gene subset are showed in the Fig 9.

## Discussion

### Heterogeneous dataset integration & expressed gene selection

Two main reasons motivate the integration of multiple gene expression datasets. Firstly, an extensive quantity of high quality samples from different platforms and technologies must be put together. This decision enriches the heterogeneity of the study, thus reinforcing its reliability and statistical robustness as well. Secondly, resulting from the previous reason, the independence of the results obtained can be guaranteed by analyzing a wider heterogeneous dataset.

The collection of a large repertoire of samples increases significantly the dimensionality, the diversity and the complexity of the experimental analysis, more so when it comes to addressing a multiclass problem. This ambitious challenge is driven by jointly analyzing multiple batches where each of them collects only a part of the classes involved in the final approach design. Table 1 reflects how the heterogeneity can be achieved by taking into account samples that have been experimentally processed at different time points, from different technologies and different platforms. Moreover, a large racial diversity can be expected given the origin of the samples. As a result of the foregoing, Table 2 includes the 678 RNA samples that were finally considered after a strict quality control phase. These samples represent 7 different cancer-related skin states from which was aimed to extract genes that may be truly representative of their manifestation. By considering several series with different number of skin states, the emergence of batch effects may become inevitable and could be seen as a possible limitation because of the partial association between series and skin states. However, in spite of the great heterogeneity that can be observed from the expression values of the 24 unprocessed series (Fig 2), a simultaneous preprocessing step across all the samples attains an homogeneous expression range (Fig 3).

In the translation from samples to genes, only those common genes that have the same coded symbol for any considered microarray platform, will appear after heterogeneous sample integration. The lack of uncommon gene symbols from different platforms is an assumed trade-off since the main purpose of this study is to integrate as many samples as possible that significantly represent each cancer-related skin state. Table 3 showed how the series from GPL96 and GPL571 Affymetrix platforms could integrate a little more than 12400 genes. This imposes a maximum number of potential genes that may eventually appear as common after the microarray integration. However, those series contain more than half of the PRIMEL samples (specifically, 73) and almost three quarters of the total NEV samples (in this case, 23). Not including those series would have had direct repercussions on the balance of classes and their representativeness in the study.

In view of this decision, a total of 9978 genes with common symbols appeared after integration and were exposed to the statistical significance process. In order to obtain genes that can become robust and reliable, very restrictive values were imposed for the statistical parameters LFC and PV. At this point, ensuring the statistical significance of the selected genes is thought to be primordial. This imposition can restrict the finding of skin cancer biomarkers that are strongly invariant against different anomalies or deviations. Under these restrictions, a small set of genes were highlighted by the tested configurations as presented in Table 4. Those genes were obtained from the intersection of configurations returning candidate biomarkers as shown in Fig 4. The final validity of the selected 17 gene set has been supported through the application of a statistical test. That test confirms the relevance of those genes to classify the 7 different cancer-related skin states versus other intrinsic factors of the heterogeneous datasets integration.

### Biological relevance of the DEGs

The relevance of these DEGs in the diagnosis of cancerous manifestations on the skin was investigated from an exhaustive search in the literature. Table 5 summarized how 11 of the 17 highlighted genes have already been strongly related to skin cancer in previous studies (ISL1, POU4F1, CLDN1, TYRP1, DSC1, TGM3, DSC3, BNC2, KRT20, LGR5 and MLANA). Regarding the 6 remaining genes, 2 of them have been linked to epithelial tissues (SOSTDC1 and SCGB2A1). The other 4 genes have not been previously highlighted as reliable biomarkers of the disease (PCP4, MYO15A, ANXA3 and CRYBA2).

Additionally, Table 7 reflects the outcome of the “Gene Set to Disease” (GS2D) text mining tool for the identified DEGs. From these results, 13 of the 17 genes in this study have been related to some pathology, disorder or disease of the skin, including the cancer-related skin states studied in this work. However, in addition to the possible relationship of the expressed genes with different cancerous manifestations and skin diseases, it is important to emphasize that 4 genes (ANXA3, LGR5, CLDN1 and KRT20) have been related to lymphatic metastasis. Similarly, 6 genes (DSC3, ISL1, TYRP1, LGR5, MYO15A and BNC2) are related to genetic predisposition to disease. In this sense, the potential relevance of these genes surpasses the scope of this study: the potential biomarkers not only reflect their relationship with different cancerous manifestations of the skin but they also seem to have some relationship with the predisposition to metastasize and to become ill.

**Table 7:** Relation between DEGs in this study and different skin diseases or disorders. A minimum number of two disease-related citations for each gene was selected, as well as one gene significantly associated at least with a disease. A maximum False Discovery Rate (FDR) equal to 0.05 was imposed.

Disease	# Genes involved	Genes (%)	LFC	PV	FDR	Gene symbols
Hutchinson’s Melanotic Freckle	1	0.06	9,4778	2,80E-03	2,10E-01	MLANA
Dermatitis Herpetiformis	1	0.06	8,4778	2,80E-03	1,05E-01	TGM3
Lentigo	1	0.06	7,4778	5,60E-03	7,00E-02	MLANA
Unknown Primary Neoplasms	1	0.06	6,4777	1,12E-02	9,31E-02	ISL1
Oculocutaneous Albinism	1	0.06	5,7778	1,81E-02	1,36E-01	TYRP1
Merkel Cell Carcinoma	1	0.06	5,3903	2,36E-02	1,47E-01	KRT20
Pemphigus	1	0.06	4,6702	3,86E-02	1,93E-01	DSC3
Vitiligo	2	0.12	4,6450	2,81E-03	7,04E-02	MLANA, TYRP1
Ichthyosis	1	0.06	4,6200	3,99E-02	1,87E-01	CLDN1
Nevus	1	0.06	4,3903	4,67E-02	1,94E-01	MLANA
Circulating Neoplastic Cells	2	0.12	4,2683	4,70E-03	7,05E-02	LGR5, KRT20
Experimental Melanoma	1	0.06	3,6702	7,58E-02	2,37E-01	TYRP1
Neuroendocrine Tumors	1	0.06	3,5472	8.23E-02	2.47E-01	ISL1
Skin Diseases	1	0.06	3,1027	1,10E-01	2,76E-01	DSC3
Basal Cell Carcinoma	1	0.06	2,6826	1,45E-01	3,11E-01	TYRP1
Atopic Dermatitis	1	0.06	2,2780	1,88E-01	3,52E-01	TGM3
Lymphatic Metastasis	4	0.25	1,6276	3,54E-02	1,90E-01	ANXA3, LGR5, CLDN1, KRT20
Skin Neoplasms	2	0.12	1,1440	2,28E-01	3,97E-01	MLANA, TYRP1
Adenoma	1	0.06	0,9561	4,08E-01	5,78E-01	LGR5
Carcinogenesis	1	0.06	0,9184	4,16E-01	5,78E-01	LGR5
Neoplasm Metastasis	1	0.06	-0,8365	8,50E-01	9,11E-01	CLDN1
Melanoma	2	0.12	0,6871	3,56E-01	5,34E-01	TYRP1, MLANA
Squamous Cell Carcinoma	3	0.19	0,6781	2,87E-01	4,79E-01	DSC3, CLDN1, TGM3
Genetic Predisposition to Disease	6	0.38	-0,6666	9,80E-01	9,93E-01	DSC3, BNC2, TYRP1, ISL1, LGR5, MYO15A
Carcinoma	1	0.06	-0,2515	7,11E-01	7,73E-01	LGR5
Neoplasm Invasiveness	3	0.19	0,2016	4,99E-01	6,24E-01	LGR5, CLDN1, KRT20

### Gene ranking assessment

Although the potential of all the identified DEGs as skin cancer biomarkers became evident, an additional evaluation of the actual information provided in a possible diagnosis test was carried out. Two additional objectives were aimed: on one hand, to further reduce the final repertoire of DEGs in order to decrease the test complexity; on the other hand, to check the potential relevance of each of the considered DEGs, especially those that were not previously related to skin cancer. The procedure followed in this study was the evaluation of different classifiers taking into account the gradual insertion of the highlighted genes according to their maximization of the discernment capability among the cancer-related skin states. From the mRMR algorithm point of view, this is translated to a gradual increase of mutual information between the identified DEGs and the skin states, avoiding as much as possible the redundancy among them.

#### Gene relevance analysis

DSC3 gene was chosen from the selected gene set as the most discriminating gen by mRMR algorithm to differentiate among the 7 cancer-related skin states. This gene, which has already been previously cataloged as skin oncogene, tends to present low gene expression levels on patients who suffer from skin melanoma (see S3 Appendix). This can be seen in Fig 7, where gene expression levels for each gene in each skin state are observed. In this gene, only its PRIMEL gene expression wide range prevents separating this skin state from the rest. Even so, DSC3 allows separating those skin states that present a greater probability of provoking malignant tumor formations and spreading (PRIMEL, METMEL and MCC) from those less aggressive or simply healthy skin states (BCC, SCC, NSK and NEV).

Following, the mRMR algorithm selected the SCGB2A1 gene as the next with more information to discern among the 7 cancer-related skin states. In this gene, at least 2 groups can be easily differentiated: 1) NSK together with MCC, and 2) the rest. It is noteworthy that this gene, that had never been related to skin cancer before, appeared in second position. However, this gene has certainly been linked to epithelial tissues and other cancers (ovary, prostate, uterus, primary and occult breast, liver, colorectal, etc.), and what it is more important, with up-expression in almost all of them. From Fig 7, we observe that in skin cancer, gene expression levels appeared down-expressed with respect to NSK for the remaining cancer-related skin states, except for MCC. In this sense, there is evidence that its gene expression level is lower for cancer-related skin states than for the other healthy skin state (NEV). For all of this, this gene could be a novel and valid biomarker that provides clues about the predisposition to suffer from some type of skin cancer. Extended information can be consulted in S3 Appendix.

BNC2 gene was ranked in third position by the feature selection algorithm. Already previously accepted as skin oncogene, this biomarker allows clearly differentiating among the 2 most diagnosed skin carcinomas (BCC and SCC). Additionally, its expression adds complementary information to what it is already provided by DSC3 and SCGB2A1, providing a better discernment among the 7 cancer-related skin states.

The gene expression differences for each of the next selected genes in the ranking can be also observed in Fig 7. It should be noted at this point that, although the mRMR ranking proposes genes with greater ability to discriminate among cancer-related skin states than others, all of them present relevant information for the specific skin states diagnosis. For example, several genes from the final part of the ranking, present specific clear information on MCC against the rest skin states as LGR5, POU4F1, SOSTDC1, KRT20, TGM3 and MYO15A genes, as their gene expression levels are opposite against to the other cancer-related skin states. Among all of them, up-expression of POU4F1 and KRT20 genes was previously related to MCC. Surprisingly, although LGR5 and TGM3 have been linked before to BCC risk, they showed here down-expressed values in MCC (Fig 7). Even going beyond, SOSTDC1 and MYO15A have not been previously reported as skin cancer biomarkers. However, they show down-expression and up-expression in MCC, respectively. On the other hand, PCP4 gene appeared as down-expressed in several skin states with respect to NSK as well as so did SCGB2A1. More biological details about these genes and their relationship with skin cancer can be seen in S3 Appendix.

#### Accuracy-complexity trade-off

Although only 17 genes fulfilled all the statistical constraints and a high overall recognition rate was obtained, there are chances that not all of them have a direct influence on improving the classifier performance. In this regard, a detailed analysis of the influence of each DEG on the classifier improvement can be made. Multiple interpretations could be drawn from the gene relevance analysis. On the one hand, it could be achieved from the interlaced analysis of their distribution on each cancer-related skin state. On the other hand, together with the previous one, it could be analysed from their influence on the classifying power of the classifier model both in the global recognition and in the specific recognition of each skin state. Thus, in search of informative power for the genes to be finally selected for the diagnosis tool, a classification accuracy improvement was assessed, by gradually adding genes from the ranking into the classifier.

The actual contribution of each gene to the classifier can be more clearly verified from the overall and specific trends in the evolution analysis seen in Figs 8 and 9. If the 17 DEGs are used, an overall accuracy above 92%, 95% and 96% can be attained when the 7, 3 and 2 classes taxonomy are used. The curves associated with each taxonomy evolve similarly for both cross-validation processes. This fact indicates that a great robustness was reached in this study from the large sample integration, which leads to the convergence of both validation processes. With respect to the 7 classes taxonomy curve trend, an ascending order is clearly observed as the genes are introduced into the classifier. Therefore, it shows that there is a gradual real information input.

Since the 3 and 2 classes taxonomies results were obtained from the 7 classes confusion matrix summary, there are certain local convergence zones in their accuracy evolutions. These events occur among the fourth and sixth genes, and from the tenth gene, from which the accuracy practically reaches its maximum value. Therefore, this quantity of genes can be considered as a suboptimal gene subset, allowing to establish a trade-off between the number of genes considered for the diagnosis model and its accuracy. Precision rounded 95.5% for 2 classes, 95% for 3 classes and 90% for 7 classes for the 10 genes model. This implies a decrease of around 2% of accuracy in the classifier performance for the main 7 classes taxonomy, at the expense of reducing in 40% the number of genes needed for diagnosing. Thus a simpler diagnosis model is possible, with the resulting economical and time reduction. To sum up, different accuracy-complexity trade-offs can be raised depending on the benefits that intend to be optimized:

1. **Minimum number of genes:** 4 DEGs, accuracies around 92% (2 classes), 90% (3 classes) and 83% (7 classes).
2. **Maximum accuracy:** All 17 DEGs, accuracies around 96% (2 classes), 95% (3 classes) and 92.5% (7 classes) (see Fig 8).
3. **Accuracy-genes trade-off approach:** 10 DEGs, accuracies around 95.5% (2 classes), 95% (3 classes) and 90% (7 classes).

Fig 9 showed how different accuracy evolutions were reached by each cancer-related skin state as the genes were gradually aggregated into the classifier model. For example, with only the first 3 genes (DSC3, SCGB2A1 and BNC2), an accuracy above 80% is insured for 4 skin states (NSK, METMEL, BCC and MCC). By selecting 10 genes as trade-off, high classification rates are reached for most cancer-related skin states: NSK (99%), PRIMEL (82%), METMEL (90%), BCC (84%), SCC (90%) and MCC (96%).

These observations suggest that different gene rankings could be returned when pursuing an optimal classification of a specific cancer-related skin state. For example, although MCC shows expression values contrary to the rest of skin states in the identified DEGs, there are genes like LGR5, POU4F1, SOSTDC1, KRT20 and MYO15A which are clearly postulated as differentiating genes in MCC diagnosing in comparison to other cancer-related skin states. However, their contribution on the MCC diagnosis improvement can not be appreciated because these genes were ranked after eleventh position and the diagnosis of this skin carcinoma does not improve after the ninth gene as can be seen in Fig 9. From the same figure, a similar conclusion can be drawn from the PCP4 gene that was ranked in tenth position and its potential informative power for diagnosing some skin state seems to be irrelevant despite having a distribution similar to SCGB2A1.

### Limitations of the approach

Although this research study has focused on offering a general view of the most relevant cancer-related skin states, there is awareness that this approach can be extended and improved in some aspects.

Human RNA samples from several skin parts were considered with the aim of getting a global and generic perspective of the disease. However, this study has dealt with the main cancerous manifestations on human skin, and specifically, only those appearing in different cells or zones located in the epidermis. According to this, other cells or skin layers (dermis, subcutaneous tissue, etc.) were not taken into account in this first approach. This was partially supported by the fact that samples concerning lymph nodes and mucosal tissues (mouth, genital areas, neck, head, etc) can introduce too specific genes that are not related to the cancerous manifestations of the most human skin outer layer.

Likewise, the consideration of skin illnesses that are categorized as skin cancer precursors such as actinic keratosis [80], psoriasis [81] or Bowen’s disease [82] were not considered and could be taken into account for future studies. Additionally, biological samples from other manufacturers (Agilent, Exon, Taqman, etc.) could be considered which would imply an increment in the sample number. For this purpose, the finding of appropriate processing techniques from platforms associated with those manufacturers for information extraction would be an immediate challenge to be assessed in future researches.

It is necessary to clarify that the aim of this pipeline is not only the integration of numerous skin samples coming from microarrays, but to co-integrate biological samples coming from other technologies. Mainly, it would be interesting to integrate with nowadays precise technologies as RNA-seq, which provides a high consistency by getting an equivalence between reads and gene expression value [21, 83, 84]. Similarly, it would be possible the integration with an older technology like quantitative polymerase chain reaction (qPCR) [85], given that multiple considerations at quality and processing level are carried out. Reverse transcription qPCR (RT-qPCR) has been considered during many years as the gold standard for measurement of gene expression [86]. Thus, and although other technologies have appeared later, Real-Time RT-qPCR can be taken into account as a suitable method for fast, accurate, sensitive and cost-effective gene expression analysis [87].

## Conclusions

Through a restrictive pipeline process, 17 DEGs were obtained for discriminating up to seven cancer-related skin states from the integration of multiple skin cancer datasets. In the light of all results and discussions presented in this work, these genes have been seen as reliable skin cancer biomarkers. Consequently, they are expected to serve as a guide to improve the early diagnosis of skin cancer because these indicate the potential predisposition to suffer from it. Many of these genes have been linked even to other pathologies or disorders of the skin that are considered as precancerous skin states.

The vast heterogeneity of the sample collection with respect to diverse factors like platforms, origin, parts of the body, etc. positively influenced in the finding of 6 genes that had not previously related to skin cancer: SCGB2A1, CRYBA2, ANXA3, PCP4, SOSTDC1 and MYO15A. In this sense, beyond the importance of each DEG in the overall recognition, the relevance analysis of each DEG showed the differentiating role of the SCGB2A1 gene. This is greatly due to the fact that the massive heterogeneous sample integration has allowed extracting extremely useful underlying information from the joint study of up to 7 different cancer-related skin states. SCGB2A1 appeared as down-expressed for all the cancer-related skin states, but MCC. The same gene was also down-expressed for the NEV state, in comparison with NSK gene expression levels. In terms of accuracy recognition, an overall recognition around 92.5% of accuracy has been achieved to distinguish among 7 cancer-related skin states. More briefly, an accuracy of 96% is guaranteed to discriminate between healthy and tumor samples from the 17 DEGs.

Our next objectives include the idea of using this pipeline in other types of cancers or diseases with a good number of existing samples from public repositories, available private data or even from further generation sequencing techniques, having data quantified in gene expression values. Additionally, modifications of the general pipeline are aimed to be used in the improvement of the diagnosis of those cancer-related skin states with lowest diagnostic accuracies.

## Supporting information

S1 Appendix Distribution of samples regarding the seven cancer-related skin states. This appendix shows the quantity of samples of each cancer-related skin state. Concretely, a table is presented where each microarray series next to high quality samples from each of them is specified.
(PDF)

S2 Appendix Detailed ANOVA test analysis. This appendix analyses and shows the full results of the performed ANOVA statistical test. This test has been carried out with the aim of determining the influence of various external and intrinsic factors on the quantification of the differentially expressed genes, when different microarray platforms and technologies are used.
(PDF)

S3 Appendix Biological interpretation of the 17 DEGs. This appendix shows detailed information about the systematic search and analysis in the scientific literature that has been published about the biological relationship of the 17 genes selected in this study, with skin and other types of cancer.
(PDF)
