# Supplementary material for: Multiclass classification for skin cancer profiling based on the integration of heterogeneous gene expression series
Source: PLoS One. 2018 May 11;13(5):e0196836. doi: 10.1371/journal.pone.0196836 (PMC5947894; doi:10.1371/journal.pone.0196836)
Supplement: S1 Appendix — This appendix shows the quantity of samples of each cancer-related skin state. Concretely, a table is presented where each microarray series next to high quality samples from each of them is specified. (PDF) [file pone.0196836.s001.pdf]

SUPPORTING INFORMATION

"Heterogeneous Data Sources Integration for Gene Expression Analysis and Multiclass Classification for Skin Cancer Profiling",

Juan Manuel Galvez, Daniel Castillo, Luis Javier Herrera, Belen San Roman, Olga Valenzuela, Francisco Manuel Ortuño, Ignacio Rojas

****

PART 1: ABOUT THE DISTRIBUTION OF THE NUMBER OF SAMPLES FOR EACH CANCER-RELATED SKIN STATE

Supplementary Table S1. RNA skin samples selected after the quality control analysis

Series	Technology	Most frequent state	Carcinoma (NMSC)			Melanoma (MSC)		Healthy Skin		TOTAL
			BCC	SCC	MCC	PRIMEL	METMEL	NSK	NEV	
GSE2503	Affymetrix	SCC		5				5		10
GSE3189	Affymetrix	PRIMEL				44		6	16	66
GSE6710	Affymetrix	NSK						12		12
GSE7553	Affymetrix	BCC	15	11		14		4		44
GSE13355	Affymetrix	NSK						57		57
GSE14905	Affymetrix	NSK						20		20
GSE15605	Affymetrix	PRIMEL				31	2	13		46
GSE29359	Illumina	METMEL					75			75
GSE30999	Affymetrix	NSK						74		74
GSE32407	Affymetrix	NSK						10		10
GSE32628	Illumina	SCC		14						14
GSE32924	Affymetrix	NSK						7		7
GSE36150	Affymetrix	MCC			10					10
GSE39612	Affymetrix	MCC	2	3	23					28
GSE42109	Affymetrix	BCC	10							10
GSE42677	Affymetrix	SCC		10						10
GSE45216	Affymetrix	SCC		28						28
GSE46517	Affymetrix	METMEL				29	35	7	7	78
GSE52471	Affymetrix	NSK						10		10
GSE53223	Affymetrix	NEV						5	9	14
GSE53462	Illumina	BCC	16	5				4		25
GSE55664	Illumina	NSK						10		10
GSE66359	Affymetrix	SCC		8						8
GSE82105	Affymetrix	NSK					6	6		12
TOTAL	Integrated		43	84	33	118	118	250	32	**678**
