# Supplementary material for: Multiclass classification for skin cancer profiling based on the integration of heterogeneous gene expression series
Source: PLoS One. 2018 May 11;13(5):e0196836. doi: 10.1371/journal.pone.0196836 (PMC5947894; doi:10.1371/journal.pone.0196836)
Supplement: S2 Appendix — This appendix analyses and shows the full results of the performed ANOVA statistical test. This test has been carried out with the aim of determining the influence of various external and intrinsic factors on the quantification of the differentially expressed genes, when different microarray platforms and technologies are used. (PDF) [file pone.0196836.s002.pdf]

SUPPORTING INFORMATION

"Heterogeneous Data Sources Integration for Gene Expression Analysis and Multiclass Classification for Skin Cancer Profiling",

Juan Manuel Galvez, Daniel Castillo, Luis Javier Herrera, Belen San Roman, Olga Valenzuela, Francisco Manuel Ortuño, Ignacio Rojas

****

PART 2: ABOUT THE STATISTICAL ANALYSIS OF THE 17 SELECTED GENES

1 Introduction to the Statistical Analysis

Our aim is to accurately determine the influence on the differential expression of genes when various factors or ways of treating the microarray are used. In addition, factors related to the type of pathology or class analyzed in this contribution will be included in this statistical study, in order to compare the statistical significance of the disease. In order to carry out this statistical analysis, a powerful technique such as ANOVA is used. Since the ANalysis Of Variance tool, often abbreviated to ANOVA, is a well-known technique, we refer to the references [1 – 4] for a detailed explanation of the ANOVA methodology.

We recall that the factors considered in the analysis are presented in Supplementary Table S2-A.

Supplementary Table S2-A. Variables used in the statistical study. All the possible configurations of factors levels

**Factors**	**Levels of the Factors**						
Country	GERMANY	NETHERLANDS	SOUTH KOREA	USA	UNITED KINGDOM	AUSTRALIA	FINLAND
Type	NEV	NSK	PRIMEL	SCC	METMEL	BCC	MCC
Batch	MRS	QD					
Method	MED	MEAN					

Due to the existence of multiple genes that are significant once the pipeline of genes selection is carried out (17 genes have been selected, which are presented in Table 5 of the manuscript), in order to perform a statistical analysis that can encompass all the information of all genes simultaneously, a dependent variable has been designed based on the concept of Least Squares (regression analysis method). This variable is defined as:

$$Avg\_distance_i = \frac{1}{N}\sum_{i=1}^{N}\sum_{j=1}^{M}\left(g_{i,j} - \hat{g}_i\right)^2$$

Where N is the number of genes used in this study (a total of 17), M is the number of measures which have been performed in the various experiments and/or pre-processing variants with these genes (a total of 2712), $g_{i,j}$ is the value of gene *i* in the experiment *j*, and $\hat{g}_i$ is therefore the average of the gene *i* in all the experiments and/or pre-processing variants:

$$\hat{g}_i = \frac{1}{M}\sum_{j=1}^{M}\left(g_{i,j}\right)^2$$

Thus for having different data from several microarray, the influence of the gene expression (over a selected set of genes) is analysed using “Avg_distance” as the dependent variable.

The next section presents the results that have been obtained in the ANOVA analysis.

2 Results of the Statistical Analysis

To carry out the statistical study, a selection is made from a set of alternatives that are representative of each of the factors being considered to perform the microarray processing. By analysing the different levels of each of these factors, it is possible to determine their influence on the performance of the analysed subset of genes when different alternatives for pre-processing the microarray and also when having different classes are presented.

Supplementary Table S2-B gives the four-way variance analysis for the whole set of processing examples of the microarray analysed in this contribution. The ANOVA table containing the sum of squares, degrees of freedom, mean square, test statistics, etc., represents the initial analysis in a compact form. This kind of tabular representation is customarily used to set out the results of the ANOVA calculations.

Supplementary Table S2-B. Analysis of Variance for dependent variable that analyze 17 genes simultaneously. The main factors marked with (*) indicated that are statistically significative.

Source (Main Factors)	*Sum of Squares*	*Df*	*Mean Square*	*F-Ratio*	*P-Value*
A:TYPE (*)	6,28	6	1,047	1521,6	**0,0000**
B:BATCH (*)	1,51	1	1,515	2202,9	**0,0000**
C:METHOD	0,001	1	0,001	1,6	**0,2005**
D:COUNTRY (*)	0,33	6	0,055	80,6	**0,0000**
RESIDUAL	1,86	2697	0,0007		
TOTAL (CORRECTED)	10,18	2711			

Of all the information presented in the ANOVA table, the major interest of the researcher will most likely be focused on the value located in the “F-Ratio” and "Sig. Level" columns. If the numbers presented in this column are less than the critical value set by the experimenter, then the effect is supposed to be significant. This value is frequently set at 0.05 and any value less than this will result in significant effects, while any value greater than previous threshold result in non-significant effects. If the effects are found to be significant using the above procedure, it implies that the means differ more than would be expected by chance alone. In terms of the above experiment, it would mean that the processing and information of the microarray were not equally effective or applicable. As can be appreciated from Supplementary Table S2-B three main factors are statistically significant: Type, Batch and Country. However, it is important to note that the factor TYPE is a very important factor (that will be analysed afterwards with the Multiple Range Test).

Thus, a detailed analysis will be performed now for each of the factors examined, using the Multiple Range Test. Supplementary Table S2-C shows the Multiple Range Test for the relevant factor TYPE in which a multiple comparison procedure is carried out to determine which means are significantly different from which others. The method currently being used to discriminate among the means is Fisher's least significant difference (LSD) procedure, and from Supplementary Table S2-C can be concluded that there are six homogenous groups (identified using columns of X's, with intersection) for the variable TYPE. These groups are: 1) NEV, 2) NSK, 3) PRIMEL and SCC, 4) SCC and METMEL, 5) BCC and finally 6) MCC. The levels of these six group are significant from a statistical point of view (analysing the behaviour of the dependent output variable) having intersection between group 3 and 5, and showing the level MCC has the highest mean value and very different for the rest, and NEV with the lowest value. This information can be graphically seen in Supplementary Figure S2-A.

Supplementary Table S2-C. Multiple Range Tests for variable TYPE

TYPE	***LS Mean***	***LS Sigma***	***Homogeneous Groups***					
NEV	0,0554275	0,00283735	X					
NSK	0,0620063	0,00175745		X				
PRIMEL	0,0738432	0,00203318			X			
SCC	0,0760163	0,00163107			X	X		
METMEL	0,0788315	0,00235225				X		
BCC	0,093826	0,00254239					X	
MCC	0,290825	0,0028085						X

Supplementary Figure S2-A. Evolution of the output variable average/mean distance by modification of the levels of factor TYPE.

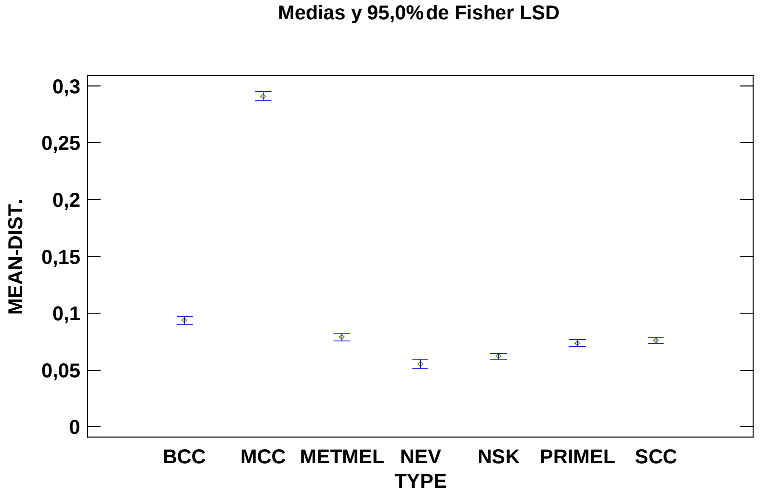

Therefore, the factor which has the most relevant impact over the expression of the selected genes is the factor that has information about the seven different cancer-related states.

A similar analysis can be performed for the other two statistically significant factor (but with less repercussion in the influence of the dependent variable).

For the variable Country, there are five homogenous groups without intersection. These groups are: 1) GERMANY, NETHERLANDS and SOUTH KOREA, 2) USA, 3) UNITED KINGDOM, 4) AUSTRALIA and finally 5) FINLAND. The levels of these five groups are completely different and significant from a statistical point of view. In this case, the level GERMANY has the lowest value and FINLAND the highest. This information can be graphically represented in Supplementary Figure S2-B.

Supplementary Table S2-D. Multiple Range Tests for variable Country

COUNTRY	***LS Mean***	***LS Sigma***	***Homogeneous Groups***				
GERMANY	0,0765293	0,00292717	X				
NETHERLANDS	0,0810193	0,00407355	X				
SOUTH KOREA	0,0812206	0,00292719	X				
USA	0,091946	0,000769304		X			
UNITED KINGDOM	0,113496	0,00323295			X		
AUSTRALIA	0,127081	0,00239614				X	
FINLAND	0,159483	0,00508002					X

Supplementary Figure S2-B. Evolution of the output variable average/mean distance by modification of the levels of factor COUNTRY.

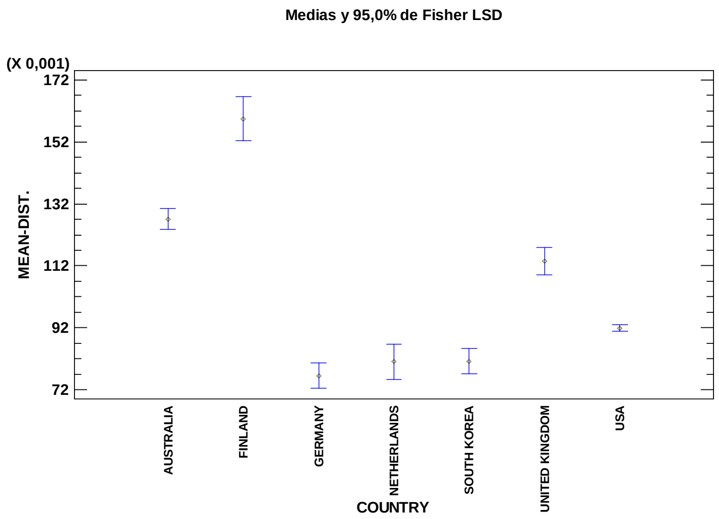

Finally, the last relevant factor is BATCH. In this case the Multiple Range Tests table is presented in Supplementary Table S2-E. There are two different groups: 1) MRS and 2) QD without intersection (Supplementary Figure S2-C).

Supplementary Table S2-E. Multiple Range Tests for variable BATCH

BATCH	***LS Mean***	***LS Sigma***	***Homogeneous Groups***	
MRS	0,0726901	0,00103097	X	
QD	0,119966	0,00103097		X

Supplementary Figure S2-C. Evolution of the output variable average/mean distance by modification of the levels of factor BATCH.

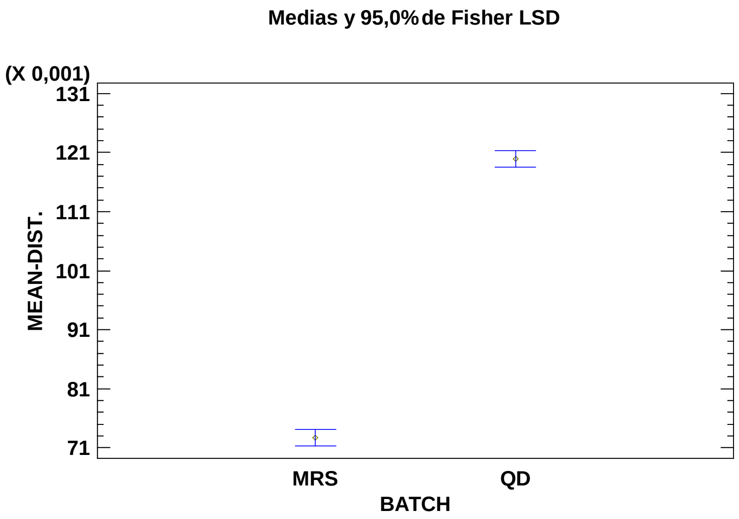

3 Results of the Statistical Analysis

From the present statistical analysis can be concluded that the factor that has a greater relevance on the behaviour or expression of the set of 17 genes that have been selected, is the factor TYPE, which represents the different skin categories selected in this contribution (skin carcinoma, skin melanoma and healthy skin categories were analysed). As it can be observed graphically, its relevance in the output variable is very remarkable, existing six different groups. Which lower repercussion than the factor TYPE, it is also relevant to mention the COUNTRY and BATCH factors, both being statistically significant among the alternatives of their levels in the output variable. Finally, the METHOD variable has no statistical significance, which means that there is no difference between pre-processing microarray selection with MED or MEAN.

Supplementary References S2

1. R.A.Fisher, Contribution to Mathematical Statistics, New York: Wiley, 1950.
2. A. Rutherford, Introducing ANOVA and ANCOVA: A GLM Approach (Introducing Statistical Methods series), Edit. Sage Publications, 2001.
3. Turner, J.R., Thayer, J., Introduction to Analysis of Variance: Design, Analysis & Interpretation Edit, Sage Publications, 2001.
4. D.C.Montgomery, Design and Analysis of Experiments, New York: Wiley. 1984.
