# Supplementary material for: Multiclass classification for skin cancer profiling based on the integration of heterogeneous gene expression series
Source: PLoS One. 2018 May 11;13(5):e0196836. doi: 10.1371/journal.pone.0196836 (PMC5947894; doi:10.1371/journal.pone.0196836)
Supplement: S3 Appendix — This appendix shows detailed information about the systematic search and analysis in the scientific literature that has been published about the biological relationship of the 17 genes selected in this study, with skin and other types of cancer. (PDF) [file pone.0196836.s003.pdf]

SUPPORTING INFORMATION

“Heterogeneous Data Sources Integration for Gene Expression Analysis and Multiclass Classification for Skin Cancer Profiling”,

Juan Manuel Galvez, Daniel Castillo, Luis Javier Herrera, Belen San Roman, Olga Valenzuela, Francisco Manuel Ortuño, Ignacio Rojas

****

PART 3: ABOUT THE BIOLOGICAL INTERPRETATION OF 17 SELECTED GENES

There are different types of skin cancer. Basal cell carcinoma (BCC) is the most common form of skin cancer while squamous cell carcinoma (SCC) is the second most common form of skin cancer [1-3]. Both squamous cell and basal cell skin carcinomas are sometimes called non-melanoma skin cancers [4]. Melanoma is the third most common form of skin cancer and is one of the most aggressive [5]. There are others less common types of skin cancer including Merkel cell carcinoma, sebaceous carcinoma, microcystic adnexal carcinoma, cutaneous lymphomas, lentigo maligna, and actinic keratosis (a pre-cancerous skin condition) [6-8].

In order to assess the relationship of the selected genes with skin cancer or their relationship with other types of cancer, we have made an analysis of published scientific literature related to these genes. Examining the literature related to these 17 genes gave insight into how these genes might be related to different types of cancer. All these selected genes are protein-coding genes. Supplementary Table S3 shows symbols and names of these genes, their function class, and the types of cancer related to these genes.

Analysis of the literature revealed that all these genes are involved in human cancer development. Furthermore, for DSC3, BNC2, TYRP1, ISL1, DSC1, MLANA, LGR5, CLDN1, POU4F1, KRT20, and TGM3 genes, we also found articles in the literature that support their association with any type of skin cancer.

In the case of desmocollins 1 and 3 (DSC3 and DSC1), a type-1 tansmembrane glycoprotreins localized in desmosomes, both types of proteins seem to be implicated in the etiology of melanoma. However, their role in cancer development and progression is not completely understood. These two desmosomal proteins have been performed both tumor suppressive and tumor promoting functions.

Supplementary Table S3. Summary of the 17 selected genes using this method.

Gene symbol	Gene name	Function class	Types of cancer related to the gene
DSC3	Desmocollin 3	Cell adhesion junctions	**Skin cancer** [10, 13]<br>Ovarian cancer [9]<br>Breast cancer [14, 15]
SCGB2A1	Secretoglobin family 2A member 1	Secreted protein	Epithelial ovarian cancer [16-19]<br>Breast cancer [20, 21]<br>Colorectal cancer [22]
BNC2	Basonuclin 2	DNA-binding zinc-finger protein	**Skin cancer**[1, 23, 24]<br>Epithelial ovarian cáncer [25-28]<br>Hepatocellular carcinoma [29]<br>Urothelial carcinomas [30]<br>Esophageal cancer [31]<br>Prostate cancer [32]
TYRP1	Tyrosinase related protein 1	Melanin synthesis	**Skin cancer** [33-41]
ISL1	ISL LIM homeobox 1	Transcription factor	**Skin cancer** [42, 43]<br>Neuroblastoma [44]<br>Gastric cancer [45]<br>Bladder cancer [46]<br>Ameloblastoma [47]<br>Soft tissue sarcomas [48]
DSC1	Desmocollin 1	Cell adhesion junctions	**Skin carcer** [49, 50]
MLANA	Melan-A	Involved in T-cell responses	**Skin cancer** [8, 51-57]
CRYBA2	Crystalline β A2	Structural components	Neuroblastoma [58]
ANXA3	Annexin A3	Ca^{2+}-regulated phospholipid- and membrane-binding proteins	Lang cancer [59]<br>Gastric cancer [60]<br>Prostate cancer [61, 62]<br>Colorectal cancer [63-65]<br>Liver cancer [66, 67]<br>Breast cancer[68]
PCP4	Purkinje cell protein 4	Regulation protein	Breast cancer [69, 70]<br>Esophageal cancer [71]
LGR5	Leucine rich repeat containing G protein-coupled receptor 5	Stem cell marker	**Skin cancer** [72-77]<br>Brain cancer [78]<br>Colon and rectum cancer [79]
CLDN1	Claudin 1	Intercellular junction	**Skin cancer** [80-86]
POU4F1	POU class homeobox 1	Transcription factor	**Skin cancer** [87-89]<br>Breast cancer [90]<br>Leukemia [91-93]<br>Cervical cancer BRN3A [94]
SOSTDC1	Sclerostin domain containing 1	Cell signaling	Lung cancer [95]<br>Thyroid cancer [96]<br>Breast cancer[97, 98]<br>Gastric cancer [99, 100]<br>Renal cancer [101, 102]<br>Epithelial ovarian cancer [103]<br>Prostate cancer [104]
KRT20	Keratin 20	Structural protein	**Skin cancer** [105-109]
TGM3	Transglutaminase 3	Enzyme	**Skin cancer** [110]<br>Oral cancer [111]<br>Esophageal cancer [112, 113]<br>Laryngeal cancer [114]
MYO15A	Myosin XVA	Myosin protein	Endocrine tumors [115, 116]

On one hand, elevated levels of DSC3, a desmosomal cadherin that is required for maintaining cell adehesions, were associated with a worse survival for melanoma [9] while Riker et al., considered DCS3 as a tumor suppressor gene due to present low level of protein expression in primary and metastatic cutaneous melanoma cell lines [10]. On the other hand, although the significance of DSC1 mutations in melanoma is unclear due to the fact that melanocytic cells do not have desmosomes [11], missense and nonsense somatic mutations were frequently found in this candidate melanoma gene [12]. Furthermore, DSC3 expression has been observed in several solid tumors. An increase of DSC3 expression has been observed in lung cancer while in other types of cancer, such as breast cancer, its expression has been reduced [13-15].

Basonuclin 2 (BNC2), a DNA-binding zinc-finger protein, is considered as a skin color gene and has been associated with SCC risk. BNC2 is expressed in melanocytes and keratinocytes, and it is thought to act both as a messenger RNA-processing enzyme and as a transcription factor [1, 23, 24]. There are many studies related to the role of BNC2 in different kinds of cancer such as epithelial ovarian cancer [25, 27, 28], hepatocellular carcinoma [29], bladder cancer [25], esophageal cancer[25, 31], urothelial carcinoma [30], and prostate cancer [32]. BNC2 expression was decreased in cancer cells, and stable expression caused cancer growth arrest. Thus, all these works suggested a putative tumor suppressor function of BNC2 during cancer development [25-32].

Tyrosinase-related protein 1 (TYRP1) is exclusively expressed in melanocytes and melanoma cells, and it is considered a pigmentation-associated gene. TYRP1 is involved in the production of eumelanin which is associated with skin fair pigmentation, being responsible for increasing skin sensitivity to sun. TYRP1 is being considered as a risk factor for the development of melanoma and a prognostic marker for metastatic skin melanoma [33-40]. Furthermore, Kosiniak-Kamysz et al. related the influence of pigmentation genes such as TYRP1 in the development of BCC [41].

The human insulin gene enhancer-binding protein islet-1 (ISL1) is a LIM-homeodomain transcription factor that promotes the proliferation of adult pancreatic islet cells. Although it is related to pancreatic neuroendocrine neoplasms, it is also related to certain groups of non-pancreatic neuroendocrine carcinomas including Merkel cell carcinoma, also called neuroendocrine carcinoma of the skin, neuroendocrine carcinomas of the lung [42, 43], and bladder cancer [46]. However, the role of ILS1 in the development of these tumors is still unclear. Furthermore, ISL1 promotes the proliferation of and gastric cancer and lymphoma cells [45] and is overexpressed in Skeletal muscle cancer [48] and ameloblastoma [47]. Besides being considered as a useful marker for metastatic pancreatic neuroendocrine neoplasms, ISL1 is also considered as a marker of neuroblastoma [44].

Protein Melan-A also known as melanoma antigen recognized by T cells 1 (MART-1)/MLANA is considered as a melanocyte marker and is important in melanoma diagnostics. Some tumors are associated with reduced expression of MLANA [51, 53]. Since its discovery in 1994, MART-1 has been the focus in the development of strategies in order to target melanoma through the immune system. In fact, it currently seems to be the most useful histological biomarker for the diagnosis of melanoma due to its nature as an specific and sensitive gene for distinguishing both primary and metastatic melanoma [52, 54, 55, 57]. MLANA is also considered to be a useful marker for identifying melanocytes in vitiligo patients' skin [56], and to confirm the diagnosis of lentigo maligna in early lesions and in the differential diagnosis from melanocytic hyperplasia in chronically sun-damaged skin [8].

Leucine-rich G-protein-coupled receptor 5 (LGR5), also known as G-protein-coupled receptor GPR49, functions as a marker of stem cells in the hair follicle in the skin, and stem cells in the small intestine and colon [72, 74-78]. LGR5 protein is involved in both SCCs [72, 73] and BCCs carcinogenesis in epidermis [74, 77], playing a significant role in tumor formation and cell proliferation. Furthermore, LGR5 is overexpressed in other types of cancer such as colorectal cancer promoting the growth of colon tumor cells [77, 79]. As expression of LGR5 is associated with activation of Wnt signaling pathway [78, 79], and Wnt signaling pathway is also involved in brain development, it is also suggested that LGR5 plays a role in maintenance and/or survival of brain cancer stem cells [78]. Thus, the overexpression of LGR5 revealed that LGR5 functions as an oncogene [77].

Claudin-1 (CLDN1) is a transmembrane protein involved in the formation of multiprotein complexes at the tight junctions in the epithelium of the skin [80, 84, 85]. Claudins regulate skin permeability, and specifically CLDN1 is suggested to be involved in proliferation and differentiation of keratinocytes. It is related to cutaneous SCC development [85, 86]. It is suggested to be downregulated by Snail and Slug (two transcription factors which promote epitheial-mesenchymal transition in malignant tumors) in cutaneous SCC [81, 85]. On the other hand, Morita et al. described a restriction of strong expression of CLDN1 to keratinized areas in 5 SCC [83]. Furthermore, CLDN1 downregulation is also implicated in the acquisition of metastatic phenotype in cutaneous melanoma [80] and in the formation of melanoma brain metastasis in brain endothelial cells. Thus, Izraely et al. suggested CLDN1 as a useful predictor for melanoma patients with a high risk of brain metastasis [82].

POU4F1 (POU class homeobox 1) is a transcription factor member of the Pit-Oct-Unc (POU) domain family, and its expression has been considered essential for melanoma cell proliferation and survival. POU4F1 has been suggested as a useful biomarker to distinguish early stage melanomas from benign lesions. 55% of human melanoma cell lines express increase levels of POU4F1, and inhibition of POU4F1 expression is involved in reducing melanoma cell viability and tumor growth. In melanoma cells, loss of POU4F1

is associated with apoptosis [87, 88]. POU4F1 has been also linked with MCC risk [89]. Furthermore, POU4F1 has been related to other cancer development such as cervical cancer [94], myeloid leukaemia [91-93], and breast cancer [90].

Cytokeratin-20 (KRT20, CK20) is an epithelial antigen that is expressed in approximately 95% of MCC thus being considered as a marker for this type of tumor [105, 107, 109]. Consequently, KRT20 expression is used as a diagnostic tool for detecting MCC [108]. However, a small amount of tumors lack KRT20 expression. This loss of KRT20 expression in cutaneous MCC has been related with decreased expression of other lineage markers such as cytokeratin 8 and chromogranin A [106].

Transglutaminase 3 (TGM3) is an enzyme with Ca2+-dependent transamidation activity in the non-proliferating layers of the epidermis. TGM3 is indispensable for normal formation of epidermis. Its principal function is catalyzing the crosslinks of proteins through the formation of isopeptide bonds between peptidyl glutamine and lysine residues. It is suggested that a genetic variant in TGM3 may disrupt the normal differentiation of corneocytes and this could increase susceptibility to BCC risk [110]. TGM3 is also down regulated in other types of cancer of head and neck [117, 118] such as oral squamous cell carcinoma [111], esophageal squamous cell carcinoma [112, 113], and laryngeal carcinoma [114].

Secretoglobin, family 2A, member 1 (SCGB2A1), also known as mammaglobin 2, is a small secreted protein of the uteroglobin superfamily. Normal expression of SCGB2A1 has been described in human ocular tissues, prostate, pituitary, and in secretory mucosal epithelia of breast, uterus and lacrimal glands [19]. SCGB2A1 overexpression has been observed in epithelial ovarian cancer [17, 18], primary epithelial breast cancer and in occult breast metastasis [17, 20, 21], liver cancer, and colorectal cancer [22]. Thus, SCGB2A1 has been reported as a predicting biomarker in several types of cancer such as epithelial ovarian cancer [16, 19], breast [17, 20], and colorectal cancer [22]. *Tassi et al*. observed that SCGB2A1 expression correlated with reduce recurrence, disease progression and death associated to cancer [19].

Sclerostin domain containing protein 1 (SOSTDC1) is an important regulator of cell signaling [101, 103]. SOSTDC1 participates in the development of various cancers acting as a tumor suppressor in several types of cancer such as gastric cancer [96, 99, 100] and non-small cell lung cancer [95]. SOSTDC1 is down regulated in epithelial ovarian cancer [103], breast cancer [95, 97, 98], gastric cancer [99, 100], renal cancer [101], prostate cancer [104], thyroid cancer [96], and non-small cell lung cancer [95]. SOSTDC1 expression is considered as a potential prognostic factor in gastric cancer [99]. SOSTDC1 down regulation has been associated with poor clinical outcome in breast cancer and high expression of SOSTDC1 has been associated with better prognosis in breast cancer [95, 97]. One way by which SOSTDC1 exert its function is by regulating both bone morphogenetic proteins (BMP) signaling pathway which are involved in activation of cell

proliferation and differentiation, and wingless/int (Wnt) signaling pathways [97, 101]. This dual regulation of BMP and Wnt signaling pathways plays a role in breast cancer [97], renal cancer [95], prostate cells [104] and kidney cells [102]. On the other hand, the tumor suppressive function of SOSTDC1 in lung cancer and thyroid cancer has been associated with the pathway that controls the activity of the retinoblastoma tumor suppressor protein (Rb), which in turn regulates the E2F transcription factor [95, 96].

Although SCGB2A1 and SOSTDC1 have not been yet linked to skin cancer, they are related to development of epithelial cancers such as ovarian and breast cancer. It is known that there are many types of epithelial cells which are cells that cover the inside and outside surfaces of the body. We may speculate that genes involved in epithelial ovarian cancer and/or epithelial breast cancer could be also involved in skin cancer development. However, the future researches will ultimately determine the validity of this approach.

Annexin 3 (ANXA3), purkinje cell protein 4 (PCP4), and myosin 15A (MYO15A) have not been related to skin cancer either. Nevertheless, all of them have been linked with certain kinds of cancer.

ANXA3 is an intracellular protein with calcium dependent phospholipid binding activity that plays a role in cellular growth and signal transduction [59, 61]. ANXA3 is considered a prognostic biomarker of lung carcinoma [59] and gastric cancer [60]. ANXA3 downregulation has been involved in prostate cancer [61, 62] and renal cancer. On the other hand, ANXA3 expression has been observed upregulated in colorectal cancer [63, 65], liver cancer [66, 67], and gastric cancer [59]. Furthermore, ANXA3 expression has been involved in proliferation, invasion and migration of breast cancer cells [68].

PCP4, also known as PEP19, is a calmodulin (CaM) binding protein that accelerates both the association and dissociation of calcium with calmodulin, regulating CaM-dependent signaling [69, 70]. PCP4 modulates calcium/CaM-dependent kinase (CaMK) activity influencing apoptosis. However, relationship between PCP4 and apoptosis has not been fully investigated yet [69]. PCP4 has been identified in studies investigating one type of esophageal cancer, squamous cell carcinoma [71]. High PCP4 expression levels have been observed in breast cancer cells where PCP4 inhibits apoptosis via CaMKK and Akt signaling pathways and increases motility [69, 70].

MYO15A is a protein that hydrolyzed adenosine triphosphate (ATP) and it is suggested to be involved in cytoplasmic organelle movement and/or hormone secretion. MYO15A is considered as a useful marker for endocrine cancers [115, 116].

Despite ANXA3, PCP4 and MYO15A have not been related to skin cancer so far, it cannot be ruled out that they will be related in the future. In fact, *Wu et al*. suggested that ANXA3 might be considered as a biological indicator for the prognosis and tumor development, invasion and metastasis [119].

Summary

Summarizing information collected from the literature, most genes identified by our approach showed association with some type of skin cancer. These results reveal the potential of our technique in selecting genes that may be considered as biomarkers for skin cancer. Further studies are necessary in order to validate these results.

Supplementary References S3

1. Chahal, H.S., et al., *Genome-wide association study identifies novel susceptibility loci for cutaneous squamous cell carcinoma.* Nat Commun, 2016. **7**: p. 12048.
2. Karia, P.S., J. Han, and C.D. Schmults, *Cutaneous squamous cell carcinoma: estimated incidence of disease, nodal metastasis, and deaths from disease in the United States, 2012.* J Am Acad Dermatol, 2013. **68**(6): p. 957-66.
3. Mohan, S.V. and A.L. Chang, *Advanced Basal Cell Carcinoma: Epidemiology and Therapeutic Innovations.* Curr Dermatol Rep, 2014. **3**: p. 40-45.
4. Pinhal, M.A., et al., *Expression of heparanase in basal cell carcinoma and squamous cell carcinoma.* An Bras Dermatol, 2016. **91**(5): p. 595-600.
5. Singh, S., et al., *Towards therapeutic advances in melanoma management: An overview.* Life Sci, 2017.
6. Connolly, K.L., K.S. Nehal, and J.J. Disa, *Evidence-Based Medicine: Cutaneous Facial Malignancies: Nonmelanoma Skin Cancer.* Plast Reconstr Surg, 2017. **139**(1): p. 181e-190e.
7. Ion, A., et al., *Proteomic Approaches to Biomarker Discovery in Cutaneous T-Cell Lymphoma.* Dis Markers, 2016. **2016**: p. 9602472.
8. Suchak, R., O.A. Hameed, and A. Robson, *Evaluation of the role of routine melan-A immunohistochemistry for exclusion of microinvasion in 120 cases of lentigo maligna.* Am J Dermatopathol, 2014. **36**(5): p. 387-91.
9. Salerno, E.P., et al., *Human melanomas and ovarian cancers overexpressing mechanical barrier molecule genes lack immune signatures and have increased patient mortality risk.* Oncoimmunology, 2016. **5**(12): p. e1240857.
10. Riker, A.I., et al., *The gene expression profiles of primary and metastatic melanoma yields a transition point of tumor progression and metastasis.* BMC Med Genomics, 2008. **1**: p. 13.
11. Haass, N.K. and M. Herlyn, *Normal human melanocyte homeostasis as a paradigm for understanding melanoma.* J Investig Dermatol Symp Proc, 2005. **10**(2): p. 153-63.
12. Nikolaev, S.I., et al., *Exome sequencing identifies recurrent somatic MAP2K1 and MAP2K2 mutations in melanoma.* Nat Genet, 2011. **44**(2): p. 133-9.
13. Chen, J., et al., *Loss of Desmocollin 3 in skin tumor development and progression.* Mol Carcinog, 2012. **51**(7): p. 535-45.
14. Klus, G.T., et al., *Down-regulation of the desmosomal cadherin desmocollin 3 in human breast cancer.* Int J Oncol, 2001. **19**(1): p. 169-74.
15. Oshiro, M.M., et al., *Epigenetic silencing of DSC3 is a common event in human breast cancer.* Breast Cancer Res, 2005. **7**(5): p. R669-80.
16. Adib, T.R., et al., *Predicting biomarkers for ovarian cancer using gene-expression microarrays.* Br J Cancer, 2004. **90**(3): p. 686-92.
17. Bellone, S., et al., *Mammaglobin B (SCGB2A1) is a novel tumour antigen highly differentially expressed in all major histological types of ovarian cancer: implications for ovarian cancer immunotherapy.* Br J Cancer, 2013. **109**(2): p. 462-71.

18. Fischer, K., et al., *Differential expression of secretoglobins in normal ovary and in ovarian carcinoma--overexpression of mammaglobin-1 is linked to tumor progression.* Arch Biochem Biophys, 2014. **547**: p. 27-36.
19. Tassi, R.A., et al., *Mammaglobin B is an independent prognostic marker in epithelial ovarian cancer and its expression is associated with reduced risk of disease recurrence.* BMC Cancer, 2009. **9**: p. 253.
20. Lacroix, M., *Significance, detection and markers of disseminated breast cancer cells.* Endocr Relat Cancer, 2006. **13**(4): p. 1033-67.
21. Zubor, P., et al., *Gene expression abnormalities in histologically normal breast epithelium from patients with luminal type of breast cancer.* Mol Biol Rep, 2015. **42**(5): p. 977-88.
22. Munakata, K., et al., *SCGB2A1 is a novel prognostic marker for colorectal cancer associated with chemoresistance and radioresistance.* Int J Oncol, 2014. **44**(5): p. 1521-8.
23. Jacobs, L.C., et al., *A Genome-Wide Association Study Identifies the Skin Color Genes IRF4, MC1R, ASIP, and BNC2 Influencing Facial Pigmented Spots.* J Invest Dermatol, 2015. **135**(7): p. 1735-42.
24. Jacobs, L.C., et al., *Comprehensive candidate gene study highlights UGT1A and BNC2 as new genes determining continuous skin color variation in Europeans.* Hum Genet, 2013. **132**(2): p. 147-58.
25. Cesaratto, L., et al., *BNC2 is a putative tumor suppressor gene in high-grade serous ovarian carcinoma and impacts cell survival after oxidative stress.* Cell Death Dis, 2016. **7**(12): p. e2526.
26. Sundqvist, J., et al., *Ovarian cancer-associated polymorphisms in the BNC2 gene among women with endometriosis.* Hum Reprod, 2011. **26**(8): p. 2253-7.
27. Wentzensen, N., et al., *Genetic variation on 9p22 is associated with abnormal ovarian ultrasound results in the Prostate, Lung, Colorectal, and Ovarian Cancer Screening Trial.* PLoS One, 2011. **6**(7): p. e21731.
28. Winham, S.J., et al., *Genome-wide investigation of regional blood-based DNA methylation adjusted for complete blood counts implicates BNC2 in ovarian cancer.* Genet Epidemiol, 2014. **38**(5): p. 457-66.
29. Wu, Y., et al., *Decreased Expression of BNC1 and BNC2 Is Associated with Genetic or Epigenetic Regulation in Hepatocellular Carcinoma.* Int J Mol Sci, 2016. **17**(2).
30. Beothe, T., D. Zubakov, and G. Kovacs, *Homozygous losses detected by array comparative genomic hybridization in multiplex urothelial carcinomas of the bladder.* Cancer Genet, 2015. **208**(9): p. 434-40.
31. Yang, Y., et al., *An integrated analysis of the effects of microRNA and mRNA on esophageal squamous cell carcinoma.* Mol Med Rep, 2015. **12**(1): p. 945-52.
32. Huang, C.N., et al., *Genetic polymorphisms in oestrogen receptor-binding sites affect clinical outcomes in patients with prostate cancer receiving androgen-deprivation therapy.* J Intern Med, 2012. **271**(5): p. 499-509.
33. Andresen, P.A., et al., *Susceptibility to Cutaneous Squamous Cell Carcinoma in Renal Transplant Recipients Associates with Genes Regulating Melanogenesis Independent of their Role in Pigmentation.* Biomark Cancer, 2013. **5**: p. 41-7.
34. El Hajj, P., et al., *SNPs at miR-155 binding sites of TYRP1 explain discrepancy between mRNA and protein and refine TYRP1 prognostic value in melanoma.* Br J Cancer, 2015. **113**(1): p. 91-8.
35. El Hajj, P., et al., *Tyrosinase-related protein 1 mRNA expression in lymph node metastases predicts overall survival in high-risk melanoma patients.* Br J Cancer, 2013. **108**(8): p. 1641-7.

36. Falkenius, J., et al., *High expression of glycolytic and pigment proteins is associated with worse clinical outcome in stage III melanoma.* Melanoma Res, 2013. **23**(6): p. 452-60.
37. Gibbs, D.C., et al., *Inherited genetic variants associated with occurrence of multiple primary melanoma.* Cancer Epidemiol Biomarkers Prev, 2015. **24**(6): p. 992-7.
38. Hertzman Johansson, C., et al., *Association of MITF and other melanosome-related proteins with chemoresistance in melanoma tumors and cell lines.* Melanoma Res, 2013. **23**(5): p. 360-5.
39. Journe, F., et al., *TYRP1 mRNA expression in melanoma metastases correlates with clinical outcome.* Br J Cancer, 2011. **105**(11): p. 1726-32.
40. Kawaguchi, M., et al., *Diacylglycerol kinase regulates tyrosinase expression and function in human melanocytes.* J Invest Dermatol, 2012. **132**(12): p. 2791-9.
41. Kosiniak-Kamysz, A., et al., *Potential association of single nucleotide polymorphisms in pigmentation genes with the development of basal cell carcinoma.* J Dermatol, 2012. **39**(8): p. 693-8.
42. Agaimy, A., et al., *ISL1 expression is not restricted to pancreatic well-differentiated neuroendocrine neoplasms, but is also commonly found in well and poorly differentiated neuroendocrine neoplasms of extrapancreatic origin.* Mod Pathol, 2013. **26**(7): p. 995-1003.
43. Huber, G.F., *Modern management of Merkel cell carcinoma.* Curr Opin Otolaryngol Head Neck Surg, 2014. **22**(2): p. 109-15.
44. Hirase, S., et al., *Early detection of tumor relapse/regrowth by consecutive minimal residual disease monitoring in high-risk neuroblastoma patients.* Oncol Lett, 2016. **12**(2): p. 1119-1123.
45. Shi, Q., et al., *ISL1, a novel regulator of CCNB1, CCNB2 and c-MYC genes, promotes gastric cancer cell proliferation and tumor growth.* Oncotarget, 2016. **7**(24): p. 36489-36500.
46. Kitchen, M.O., et al., *Methylation of HOXA9 and ISL1 Predicts Patient Outcome in High-Grade Non-Invasive Bladder Cancer.* PLoS One, 2015. **10**(9): p. e0137003.
47. Heikinheimo, K., et al., *Early dental epithelial transcription factors distinguish ameloblastoma from keratocystic odontogenic tumor.* J Dent Res, 2015. **94**(1): p. 101-11.
48. Erlenbach-Wunsch, K., et al., *Expression of the LIM homeobox domain transcription factor ISL1 (Islet-1) is frequent in rhabdomyosarcoma but very limited in other soft tissue sarcoma types.* Pathology, 2014. **46**(4): p. 289-95.
49. Gyongyosi, E., et al., *Transcriptional regulation of genes involved in keratinocyte differentiation by human papillomavirus 16 oncoproteins.* Arch Virol, 2015. **160**(2): p. 389-98.
50. Jurcic, V., J. Kukovic, and N. Zidar, *Expression of desmosomal proteins in acantholytic squamous cell carcinoma of the skin.* Histol Histopathol, 2015. **30**(8): p. 945-53.
51. Bonnelykke-Behrndtz, M.L., et al., *MelanA-negative spindle-cell associated melanoma, a distinct inflammatory phenotype correlated with dense infiltration of CD163 macrophages and loss of E-cadherin.* Melanoma Res, 2015. **25**(2): p. 113-8.
52. Danga, M.E., R. Yaar, and J. Bhawan, *Melan-A positive dermal cells in malignant melanoma in situ.* J Cutan Pathol, 2015. **42**(6): p. 388-93.
53. De Luca, T., et al., *miR-211 and MITF modulation by Bcl-2 protein in melanoma cells.* Mol Carcinog, 2016. **55**(12): p. 2304-2312.
54. Weide, B., et al., *Myeloid-derived suppressor cells predict survival of patients with advanced melanoma: comparison with regulatory T cells and NY-ESO-1- or melan-A-specific T cells.* Clin Cancer Res, 2014. **20**(6): p. 1601-9.
55. Zubovits, J., et al., *HMB-45, S-100, NK1/C3, and MART-1 in metastatic melanoma.* Hum Pathol, 2004. **35**(2): p. 217-23.

56. Kubanov, A., et al., *Immunohistochemical analysis of melanocyte content in different zones of vitiligo lesions using the Melan-A marker.* Acta Dermatovenerol Alp Pannonica Adriat, 2016. **25**(1): p. 5-9.
57. Najjar, Y.G., et al., *Melanoma antigen-specific effector T cell cytokine secretion patterns in patients treated with ipilimumab.* J Transl Med, 2017. **15**(1): p. 39.
58. Abe, M., et al., *Identification of genes targeted by CpG island methylator phenotype in neuroblastomas, and their possible integrative involvement in poor prognosis.* Oncology, 2008. **74**(1-2): p. 50-60.
59. Liu, Y.F., et al., *Annexin A3 Knockdown Suppresses Lung Adenocarcinoma.* Anal Cell Pathol (Amst), 2016. **2016**: p. 4131403.
60. Wang, K. and J. Li, *Overexpression of ANXA3 is an independent prognostic indicator in gastric cancer and its depletion suppresses cell proliferation and tumor growth.* Oncotarget, 2016. **7**(52): p. 86972-86984.
61. Penzvalto, Z., et al., *Identifying resistance mechanisms against five tyrosine kinase inhibitors targeting the ERBB/RAS pathway in 45 cancer cell lines.* PLoS One, 2013. **8**(3): p. e59503.
62. Peraldo-Neia, C., et al., *Epidermal Growth Factor Receptor (EGFR) mutation analysis, gene expression profiling and EGFR protein expression in primary prostate cancer.* BMC Cancer, 2011. **11**: p. 31.
63. Chang, Y.T., et al., *Verification of gene expression profiles for colorectal cancer using 12 internet public microarray datasets.* World J Gastroenterol, 2014. **20**(46): p. 17476-82.
64. Uzozie, A.C., et al., *Targeted proteomics for multiplexed verification of markers of colorectal tumorigenesis.* Mol Cell Proteomics, 2017.
65. Uzozie, A.C., et al., *Targeted Proteomics for Multiplexed Verification of Markers of Colorectal Tumorigenesis.* Mol Cell Proteomics, 2017. **16**(3): p. 407-427.
66. Pan, Q.Z., et al., *Annexin A3 as a potential target for immunotherapy of liver cancer stem-like cells.* Stem Cells, 2015. **33**(2): p. 354-66.
67. Pan, Q.Z., et al., *Annexin A3 promotes tumorigenesis and resistance to chemotherapy in hepatocellular carcinoma.* Mol Carcinog, 2015. **54**(8): p. 598-607.
68. Zhou, T., et al., *Silencing of ANXA3 expression by RNA interference inhibits the proliferation and invasion of breast cancer cells.* Oncol Rep, 2017. **37**(1): p. 388-398.
69. Hamada, T., et al., *Anti-apoptotic effects of PCP4/PEP19 in human breast cancer cell lines: a novel oncotarget.* Oncotarget, 2014. **5**(15): p. 6076-86.
70. Yoshimura, T., et al., *PCP4/PEP19 promotes migration, invasion and adhesion in human breast cancer MCF-7 and T47D cells.* Oncotarget, 2016. **7**(31): p. 49065-49074.
71. Visser, E., et al., *Prognostic gene expression profiling in esophageal cancer: a systematic review.* Oncotarget, 2017. **8**(3): p. 5566-5577.
72. Cammareri, P., et al., *Inactivation of TGFbeta receptors in stem cells drives cutaneous squamous cell carcinoma.* Nat Commun, 2016. **7**: p. 12493.
73. da Silva-Diz, V., et al., *Progeny of Lgr5-expressing hair follicle stem cell contributes to papillomavirus-induced tumor development in epidermis.* Oncogene, 2013. **32**(32): p. 3732-43.
74. Jia, J., et al., *LGR5 expression is controled by IKKalpha in basal cell carcinoma through activating STAT3 signaling pathway.* Oncotarget, 2016. **7**(19): p. 27280-94.
75. Liu, S., et al., *Ginsenoside rh2 inhibits cancer stem-like cells in skin squamous cell carcinoma.* Cell Physiol Biochem, 2015. **36**(2): p. 499-508.
76. Liu, S., et al., *Lgr5-positive cells are cancer stem cells in skin squamous cell carcinoma.* Tumour Biol, 2014. **35**(11): p. 11605-12.
77. Tanese, K., et al., *G-protein-coupled receptor GPR49 is up-regulated in basal cell carcinoma and promotes cell proliferation and tumor formation.* Am J Pathol, 2008. **173**(3): p. 835-43.

78. Nakata, S., et al., *LGR5 is a marker of poor prognosis in glioblastoma and is required for survival of brain cancer stem-like cells.* Brain Pathol, 2013. **23**(1): p. 60-72.
79. Takahashi, H., et al., *Significance of Lgr5(+ve) cancer stem cells in the colon and rectum.* Ann Surg Oncol, 2011. **18**(4): p. 1166-74.
80. Cohn, M.L., et al., *Loss of claudin-1 expression in tumor-associated vessels correlates with acquisition of metastatic phenotype in melanocytic neoplasms.* J Cutan Pathol, 2005. **32**(8): p. 533-6.
81. Hintsala, H.R., et al., *Claudins 1, 2, 3, 4, 5 and 7 in solar keratosis and squamocellular carcinoma of the skin.* Int J Clin Exp Pathol, 2013. **6**(12): p. 2855-63.
82. Izraely, S., et al., *The metastatic microenvironment: Claudin-1 suppresses the malignant phenotype of melanoma brain metastasis.* Int J Cancer, 2015. **136**(6): p. 1296-307.
83. Morita, K., S. Tsukita, and Y. Miyachi, *Tight junction-associated proteins (occludin, ZO-1, claudin-1, claudin-4) in squamous cell carcinoma and Bowen's disease.* Br J Dermatol, 2004. **151**(2): p. 328-34.
84. Ouban, A., et al., *Claudin-1 expression in squamous cell carcinomas of different organs: comparative study of cancerous tissues and normal controls.* Int J Surg Pathol, 2012. **20**(2): p. 132-8.
85. Rachow, S., et al., *Occludin is involved in adhesion, apoptosis, differentiation and Ca2+-homeostasis of human keratinocytes: implications for tumorigenesis.* PLoS One, 2013. **8**(2): p. e55116.
86. Ratovitski, E.A., *Phospho-DeltaNp63alpha regulates AQP3, ALOX12B, CASP14 and CLDN1 expression through transcription and microRNA modulation.* FEBS Lett, 2013. **587**(21): p. 3581-6.
87. Besch, R. and C. Berking, *POU transcription factors in melanocytes and melanoma.* Eur J Cell Biol, 2014. **93**(1-2): p. 55-60.
88. Hohenauer, T., et al., *The neural crest transcription factor Brn3a is expressed in melanoma and required for cell cycle progression and survival.* EMBO Mol Med, 2013. **5**(6): p. 919-34.
89. Leonard, J.H., et al., *Proneural and proneuroendocrine transcription factor expression in cutaneous mechanoreceptor (Merkel) cells and Merkel cell carcinoma.* Int J Cancer, 2002. **101**(2): p. 103-10.
90. Faryna, M., et al., *Genome-wide methylation screen in low-grade breast cancer identifies novel epigenetically altered genes as potential biomarkers for tumor diagnosis.* FASEB J, 2012. **26**(12): p. 4937-50.
91. Dunne, J., et al., *AML1/ETO proteins control POU4F1/BRN3A expression and function in t(8;21) acute myeloid leukemia.* Cancer Res, 2010. **70**(10): p. 3985-95.
92. Dunne, J., et al., *AML1/ETO and POU4F1 synergy drives B-lymphoid gene expression typical of t(8;21) acute myeloid leukemia.* Leukemia, 2012. **26**(5): p. 1131-5.
93. Fortier, J.M., et al., *POU4F1 is associated with t(8;21) acute myeloid leukemia and contributes directly to its unique transcriptional signature.* Leukemia, 2010. **24**(5): p. 950-7.
94. Das Purkayastha, B.P. and J.K. Roy, *Molecular analysis of oncogenicity of the transcription factor, BRN3A, in cervical cancer cells.* J Cancer Res Clin Oncol, 2011. **137**(12): p. 1859-67.
95. Liu, L., et al., *SOSTDC1 is down-regulated in non-small cell lung cancer and contributes to cancer cell proliferation.* Cell Biosci, 2016. **6**: p. 24.
96. Liang, W., et al., *Down-regulation of SOSTDC1 promotes thyroid cancer cell proliferation via regulating cyclin A2 and cyclin E2.* Oncotarget, 2015. **6**(31): p. 31780-91.

97. Clausen, K.A., et al., *SOSTDC1 differentially modulates Smad and beta-catenin activation and is down-regulated in breast cancer.* Breast Cancer Res Treat, 2011. **129**(3): p. 737-46.
98. Rawat, A., G. Gopisetty, and R. Thangarajan, *E4BP4 is a repressor of epigenetically regulated SOSTDC1 expression in breast cancer cells.* Cell Oncol (Dordr), 2014. **37**(6): p. 409-19.
99. Gopal, G., et al., *SOSTDC1 down-regulation of expression involves CpG methylation and is a potential prognostic marker in gastric cancer.* Cancer Genet, 2013. **206**(5): p. 174-82.
100. Rajkumar, T., et al., *Identification and validation of genes involved in gastric tumorigenesis.* Cancer Cell Int, 2010. **10**: p. 45.
101. Blish, K.R., et al., *Loss of heterozygosity and SOSTDC1 in adult and pediatric renal tumors.* J Exp Clin Cancer Res, 2010. **29**: p. 147.
102. Blish, K.R., et al., *A human bone morphogenetic protein antagonist is down-regulated in renal cancer.* Mol Biol Cell, 2008. **19**(2): p. 457-64.
103. Ju, W., et al., *Identification of genes with differential expression in chemoresistant epithelial ovarian cancer using high-density oligonucleotide microarrays.* Oncol Res, 2009. **18**(2-3): p. 47-56.
104. Tesfay, L., et al., *Hepcidin regulation in prostate and its disruption in prostate cancer.* Cancer Res, 2015. **75**(11): p. 2254-63.
105. Aljufairi, E. and F. Alhilli, *Merkel Cell Carcinoma Arising in an Epidermal Cyst.* Am J Dermatopathol, 2016.
106. Harms, P.W., et al., *Next generation sequencing of Cytokeratin 20-negative Merkel cell carcinoma reveals ultraviolet-signature mutations and recurrent TP53 and RB1 inactivation.* Mod Pathol, 2016. **29**(3): p. 240-8.
107. Pulitzer, M.P., et al., *Cutaneous squamous and neuroendocrine carcinoma: genetically and immunohistochemically different from Merkel cell carcinoma.* Mod Pathol, 2015. **28**(8): p. 1023-32.
108. Saini, A.T. and B.A. Miles, *Merkel cell carcinoma of the head and neck: pathogenesis, current and emerging treatment options.* Onco Targets Ther, 2015. **8**: p. 2157-67.
109. Tsai, Y.Y., et al., *CK7+/CK20- Merkel cell carcinoma presenting as inguinal subcutaneous nodules with subsequent epidermotropic metastasis.* Acta Derm Venereol, 2010. **90**(4): p. 438-9.
110. Stacey, S.N., et al., *Germline sequence variants in TGM3 and RGS22 confer risk of basal cell carcinoma.* Hum Mol Genet, 2014. **23**(11): p. 3045-53.
111. Choi, P., et al., *Examination of oral cancer biomarkers by tissue microarray analysis.* Arch Otolaryngol Head Neck Surg, 2008. **134**(5): p. 539-46.
112. Liu, W., et al., *Functional studies of a novel oncogene TGM3 in human esophageal squamous cell carcinoma.* World J Gastroenterol, 2006. **12**(24): p. 3929-32.
113. Uemura, N., et al., *Transglutaminase 3 as a prognostic biomarker in esophageal cancer revealed by proteomics.* Int J Cancer, 2009. **124**(9): p. 2106-15.
114. Liu, J., et al., *[Expression of TGM3 protein and its significance in laryngeal carcinoma].* Lin Chung Er Bi Yan Hou Tou Jing Wai Ke Za Zhi, 2012. **26**(3): p. 101-3.
115. La Rosa, S., C. Capella, and R.V. Lloyd, *Localization of myosin XVA in endocrine tumors of gut and pancreas.* Endocr Pathol, 2002. **13**(1): p. 29-37.
116. Lloyd, R.V., et al., *Myosin XVA expression in the pituitary and in other neuroendocrine tissues and tumors.* Am J Pathol, 2001. **159**(4): p. 1375-82.
117. Nair, J., et al., *Gene and miRNA expression changes in squamous cell carcinoma of larynx and hypopharynx.* Genes Cancer, 2015. **6**(7-8): p. 328-40.
118. Wu, X., et al., *TGM3, a candidate tumor suppressor gene, contributes to human head and neck cancer.* Mol Cancer, 2013. **12**(1): p. 151.

119. Wu, N., et al., *The role of annexin A3 playing in cancers.* Clin Transl Oncol, 2013. **15**(2): p. 106-10.
